# The Effects of Different Feeding Routines on Welfare in Laboratory Mice

**DOI:** 10.3389/fvets.2019.00479

**Published:** 2020-01-14

**Authors:** Janina Feige-Diller, Viktoria Krakenberg, Louisa Bierbaum, Leonie Seifert, Rupert Palme, Sylvia Kaiser, Norbert Sachser, S. Helene Richter

**Affiliations:** ^1^Department of Behavioural Biology, University of Münster, Münster, Germany; ^2^DFG Research Training Group EvoPAD, University of Münster, Münster, Germany; ^3^Department of Biomedical Sciences, University of Veterinary Medicine, Vienna, Austria

**Keywords:** feeding routines, welfare, laboratory mice, anxiety-like behavior, corticosterone metabolites, body weight

## Abstract

The accepted norm in most laboratories around the globe is feeding laboratory mice an *ad libitum* diet, although several health impairments are well-established. In contrast, reducing the animals' body weight by feeding them less food once per day (referred to as 24 h schedule) has been shown to enhance life span and reduce disease susceptibility. Against this background, this study aimed at systematically investigating the effects of different feeding routines. Therefore, three feeding routines were compared to the standard *ad libitum* feeding and effects on body weight development and welfare were investigated in male C57BL/6J mice. In particular, a 24 h schedule group, an AUTO group, characterized by an automated supply of small pieces of food all over the day, and a 4 h removal group, characterized by daily removal of food for 4 h, were studied. While the removal of food for 4 h per day did not lead to a reduction of body weight, and hence is unlikely to prevent negative effects of overfeeding, both the 24 h schedule group and the AUTO group led to the aspired body weight reduction. In the AUTO group, however, higher levels of corticosterone metabolites and stereotypies were observed, implying a rather negative impact on welfare. By contrast, no distinct negative effects of a 24 h schedule were found. Studies like this underline the general need for evidence-based severity assessments of any procedure involving living animals.

## Introduction

The laboratory mouse is the most frequently used model organism for diverse research questions worldwide. Notably, in Germany alone, ~1.4 million mice were used for research purposes in 2017 (1). To help ensure the humane use of animals, the “3R-concept,” first described by Russell and Burch (2), has gained a central role in laboratory animal science. It encompasses the replacement, reduction and refinement of animal experiments wherever possible. Refinement, in particular, has the goal of reducing the suffering of animals in research and of improving their general welfare. In this context, severity assessment is currently one of the major aims in refinement research.

When considering the severity of animal research, emphasis has mostly been put on the evaluation of experimental procedures. However, refining housing routines is presumably as important for welfare as refining experimental techniques [e.g., see (3, 4)]. In this respect, meeting species-specific nutritional demands may represent one of the most central factors in an animal's life that contributes to good welfare. Accordingly, the current European Directive 2010/63/EU^1^ clearly states that “[t]he form, content and presentation of the diet shall meet the nutritional and behavioral needs of the animal.” Yet, so far, little research has been done on what exactly this means for a laboratory mouse.

Looking at the wild house mouse, individuals spend a large proportion of their time on foraging at 20–30 different food sites every night, which can vary profoundly in the nutritional value of the respective food [reviewed by Latham and Mason (5)]. By contrast, the accepted “norm” in most laboratories around the globe is feeding an *ad libitum* diet. Although these diets are specifically designed as maintenance food for rodents, they are characterized by access to high-energy food around the clock presented in the food tray of the cage. This method prevents contamination of the food with feces or urine and is easy to include in the everyday housing routines. However, it is not only fundamentally different to the natural foraging behavior of house mice, but also comes along with several well-known adverse effects on health and well-being. In particular, *ad libitum* feeding has been shown to cause obesity and high levels of body fat in laboratory mice and rats (6, 7). Furthermore, it has been linked to early mortality due to severe degenerative disease and to a higher incidence of spontaneous tumors (8–10).

However, despite this evidence, other feeding routines, such as the intermittent provision of food, are hardly ever applied in laboratory practice. Solely in the context of specific experimental techniques, alternative feeding routines come into play, for example to guarantee a high motivation of the animals to participate in operant tasks [reviewed by Rowland (11), Toth and Gardiner (12)]. In these cases, mice are often fed according to a 24 h schedule, receiving a reduced amount of food of the same diet and thus a reduced amount of metabolizable energy once per day, so that the body weight is decreased by a certain percentage of the initial *ad libitum* weight [e.g., (13–18)]. Although this procedure usually does not aim at improving the animals' welfare, there is evidence that it prevents from the aforementioned negative effects of *ad libitum* feeding [reviewed by Mattson (19); see also (20–22)]. Furthermore, positive effects of bodyweight reductions on survival have been found (23). Likewise, a negative correlation has been shown for the average daily food consumption and the survival of *ad libitum* fed rats (24). These studies suggest that the observed health benefits of a 24 h schedule most likely result from the decrease in body weight rather than the change in food delivery. Further positive effects include an increased learning ability and memory in young animals (25) and a reduced age-dependent decline of cognitive or locomotor functions (26). Against this background, the aim of the present study was to systematically compare different feeding routines regarding their effect on body weight development and animal welfare. In two separate experiments, male C57BL/6J mice were fed according to one of four feeding routines. In a first experiment, *ad libitum* feeding was compared to a 24 h schedule and a 4 h removal group. The latter tested, whether the removal of food for 4 h daily in a period of high feeding activity was sufficient to downregulate body weights to similar levels as seen in a 24 h schedule. In the second experiment, one group of mice received a reduced amount of food as several small pellets across the day supplied by an automated feeding device (AUTO group). Thereby the natural feeding behavior of eating several small meals per day was mimicked and again compared to *ad libitum* feeding and a 24 h schedule.

To gain a picture as comprehensive as possible of the individuals' welfare, body weight development and several well-established welfare measures [e.g., see (27, 28)] were systematically investigated. In particular, fecal corticosterone metabolites, anxiety-like and exploratory behavior, spatial learning abilities, nesting behavior as well as home cage behavior were studied. This way, the hypothesis was tested that the three alternative feeding routines differ in their effect on body weight development and the animals' welfare from *ad libitum* feeding.

## Materials and Methods

### Animals and Housing Conditions

The study was performed in two independent experiments, each involving 36 male C57BL/6J mice, obtained from Charles River Laboratories (Research Models and Services, Germany GmbH, Sulzfeld). After arrival in our institute, mice were kept in groups of three to four animals in transparent standard Makrolon type III cages (38 × 22 × 15 cm) with *ad libitum* access to food (Altromin 1324, Altromin GmbH, Lage, Germany) and tap water. The cages were equipped with wood shavings as bedding material (Exp. 1: Allspan Olympia-Einstreu, Allspan Spanverarbeitung GmbH, Karlsruhe, Germany; Exp. 2: Tierwohl, J. Reckhorn GmbH & Co.KG, Rosenberg, Germany) and a paper towel as nesting material. Further enrichment was provided by a transparent red plastic mouse house (Mouse House™, Tecniplast Deutschland GmbH, Hohenpeißenberg, Germany) as a hiding possibility and a wooden stick for gnawing. Individual earmarks were used to identify all animals. Before the start of the experimental phase at full adulthood (PND 76), all mice were transferred to single housing conditions to avoid any aggression between group-housed male mice. Cages were changed biweekly and positions of the cages in the housing room were balanced across the treatments. The housing room was set to a 12 h light/dark cycle with lights off at 8 a.m., a temperature of about 22°C and a relative air humidity of about 50%.

### Ethics Statement

All procedures complied with the regulations covering animal experimentation within Germany (Animal Welfare Act) and the EU (European Communities Council DIRECTIVE 2010/63/EU)^1^ and were approved by the local (Gesundheits-und Veterinäramt Münster, Nordrhein-Westfalen) and federal authorities (Landesamt für Natur, Umwelt und Verbraucherschutz Nordrhein-Westfalen “LANUV NRW”).

### Experimental Design

Both experiments aimed to compare different feeding routines to investigate the consequences on the welfare of laboratory mice. In the respective experiments, mice were randomly assigned to one of three treatment groups (*n* = 12 per group). After reaching full adulthood and prior to the start of the different feeding routines at PND 76, the individuals were weighed on a daily basis for at least five consecutive days [e.g., (18)] using a digital scale (accuracy: 0.1 g; CM 150-1N, Kern, Balligen, Germany). The maximum weight during this period was set as their reference initial body weight. A typical maintenance diet for laboratory rodents (Altromin 1324, Altromin GmbH, Lage, Germany) with a metabolizable energy content of ~3,227 kcal/kg and 11% fat, 24% protein, and 65% carbohydrates was used for all feeding routines. All experimental procedures were conducted by experienced researchers (LS, LB, JF-D).

#### Experiment 1

**Figure 1:**
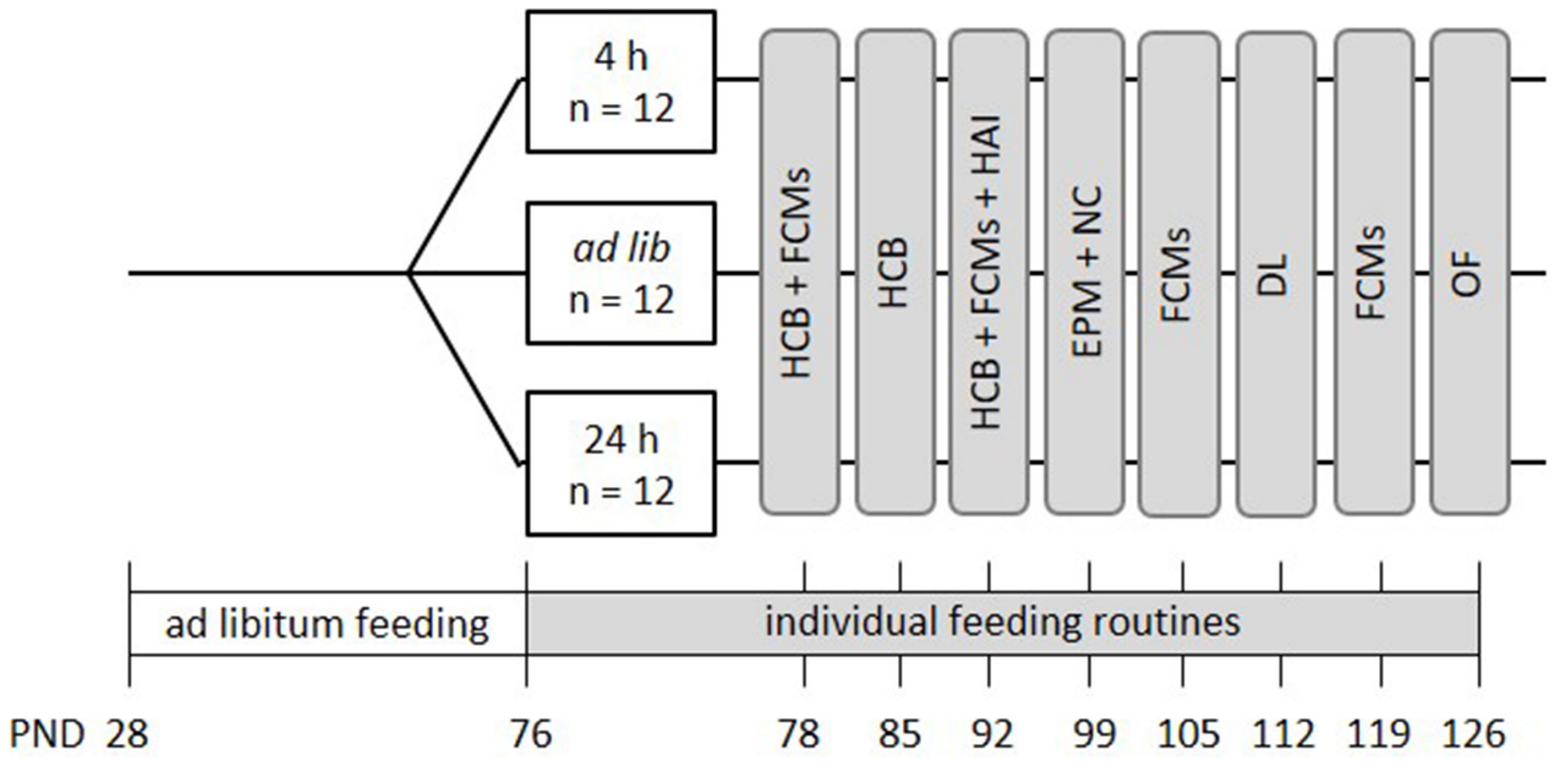
Schematic timeline of experiment 1. From PND 76 on, the animals were subjected to individual feeding routines and either fed *ad libitum* (*ad lib*), according to the 4 h removal (4 h) or the 24 h schedule (24 h). They were kept to this diet for a total experimental time of 8 weeks. Effects on animal welfare were evaluated by home cage behavior observations (HCB), measurements of fecal corticosterone metabolites (FCMs), and different behavioral tests, including the Human-Animal Interaction Test (HAI), the Novel Cage Test (NC), the Elevated Plus Maze Test (EPM), the Dark-Light Test (DL) and the Open Field Test (OF). All information on PNDs have an accuracy of ±2 days. Please note that in this experiment no base values of corticosterone metabolite concentrations were assessed.

In this project, the following three feeding routines were compared: *ad libitum* feeding, feeding a specific amount of food once per day (24 h schedule) and free access to food except during 4 h per day (4 h removal, Figure 1). Animals of the 24 h schedule received one big food pellet once per day directly after weighing. The size of the pellet was adjusted on a daily basis to reduce the animal's weight to 90–95% of its initial body weight. On average, mice of the 24 h schedule received 3.4 g of food each day. The 4 h removal group had free access to food except during the time from 8 a.m. to 12 p.m., which is the time directly after the lights were turned off. Body weights of all groups were assessed subsequent to the food removal in the 4 h removal group, daily between 11 a.m. and 12:30 p.m. Thereby, it was ensured that all animals were handled only in the dark, i.e., their active phase.

Mice were kept to the different feeding routines for a total duration of 8 weeks (Figure 1). During this experimental time, a series of tests was conducted to evaluate the effects of the feeding routines on the animals' welfare. Briefly, fecal corticosterone metabolites (FCMs) were measured non-invasively every other week (PND = 78 ± 1, 92 ± 1, 105, 119, Figure 1). Furthermore, home cage observations were conducted during the first 3 weeks of the experimental phase to record basic activity levels of the animals (HCB; PND = 76-93, Figure 1). Finally, a battery of behavioral tests was conducted between PNDs 94 and 127, including the Human-Animal Interaction test (HAI), the Elevated Plus Maze (EPM), Novel Cage Test (NC), Dark Light Test (DL), and the Open Field Test (OF) to investigate human-animal interactions, anxiety-like behavior and exploratory locomotion (for details see Figure 1).

#### Experiment 2

**Figure 2:**
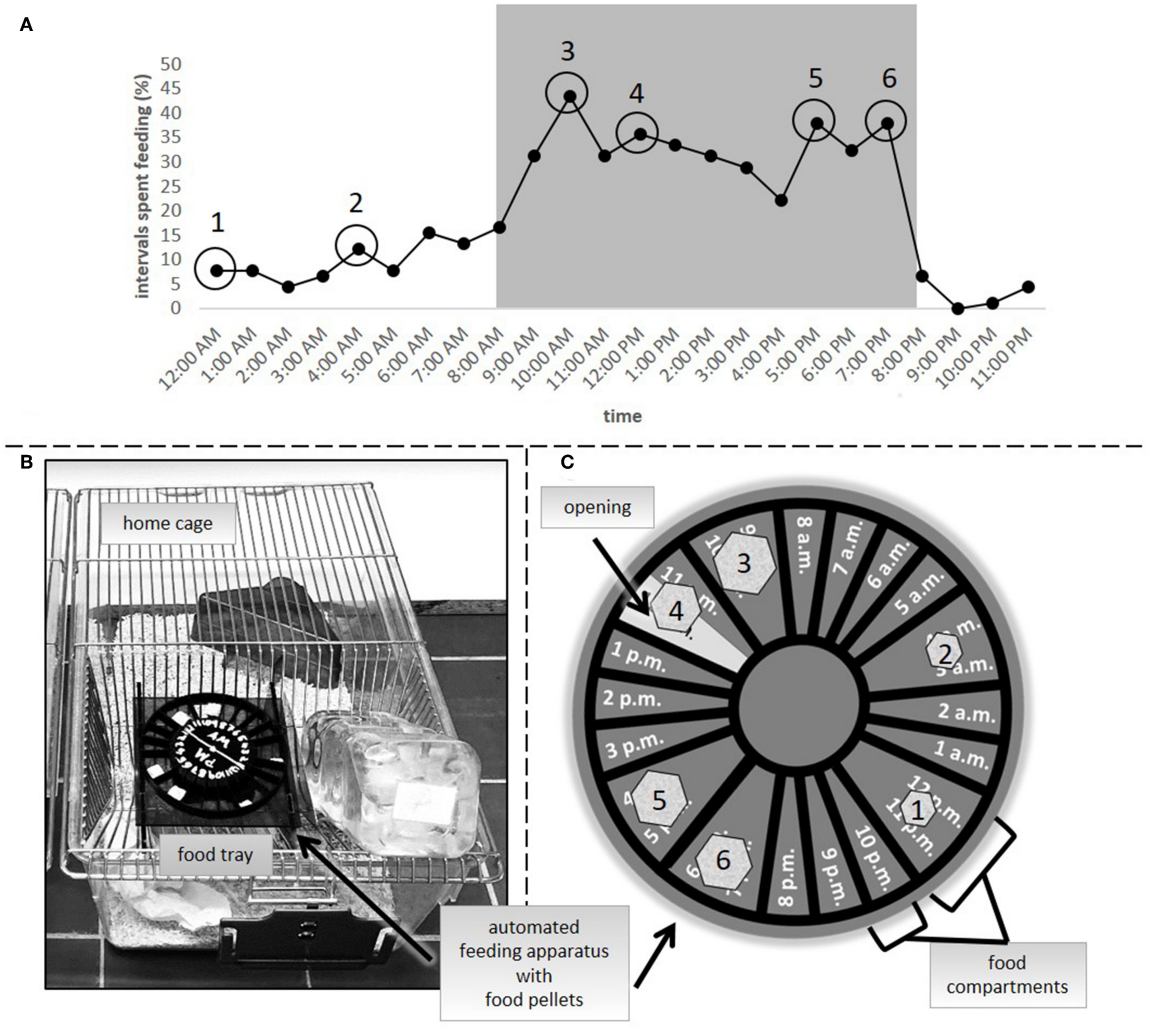
**(A)** Feeding activity as percentage of intervals spent feeding in *ad libitum* fed mice (*n* = 15). Numbers 1–6 symbolize the feeding time points in the AUTO group. **(B)** Clock-like apparatus positioned in the food tray of a home cage. **(C)** Symbolic representation of the automated feeding apparatus. Food pellets were divided into smaller pieces, reflecting differences in the feeding activity at the different time points [1–6, as indicated in **(A)**] and placed into the respective food compartments.

In the second experiment, three groups were compared: *ad libitum* feeding, a 24 h schedule and a group in which food was automatically delivered by a clock-like apparatus that was placed onto the cage lid [automated feeding routine, AUTO, adapted from SnackClock (KravitzLab)]. Both the *ad libitum* group and the 24 h schedule group were treated in the same way as in experiment 1. Similar to a 24 h schedule, the amount of food for animals of the AUTO group was adjusted on a daily basis to reduce their body weight to 90–95% of their reference initial body weight. On average, mice of the 24 h schedule and the AUTO group received about 3.5 g of food each day. The apparatus which delivered the food pellets in the AUTO group consisted of a clockwork to which a wheel-like structure was attached (Figures 2B,C). The wheel included several small compartments for pellets, which would fall into the food tray at specific time points spread over the day. A complete turn of the wheel equaled 24 h. To determine the specific feeding time points, 15 mice were video recorded (EH1000H- 4 Nano cameras, AVer Information Inc., Taiwan) for 72 h after they were transferred to individual housing conditions (PND = 62 to 66). Subsequently, their feeding profile was analyzed using instantaneous sampling every 10 min (Figure 2A). Feeding behavior was defined according to previous publications (29). The profile was used for choosing six feeding time points according to the observed feeding pattern. Four time points of high feeding activity were chosen for the dark period (10 a.m., 12 p.m., 5 p.m., and 7 p.m.). Since general feeding activity was low during the light period, for this time only two time points were chosen (12 a.m. and 4 a.m.). For the calculation of food amount, body weights were assessed daily before the first delivery time point in the AUTO group between 8 and 9:30 a.m. Therefore, the time of body weight assessment differed slightly from experiment 1.The 24 h schedule group received their daily adjusted food amount as one big pellet per day. The food for animals of the AUTO group was divided into six smaller parts, relative to the feeding activity recorded at the different time points, and placed into the respective food compartment.

**Figure 3:**
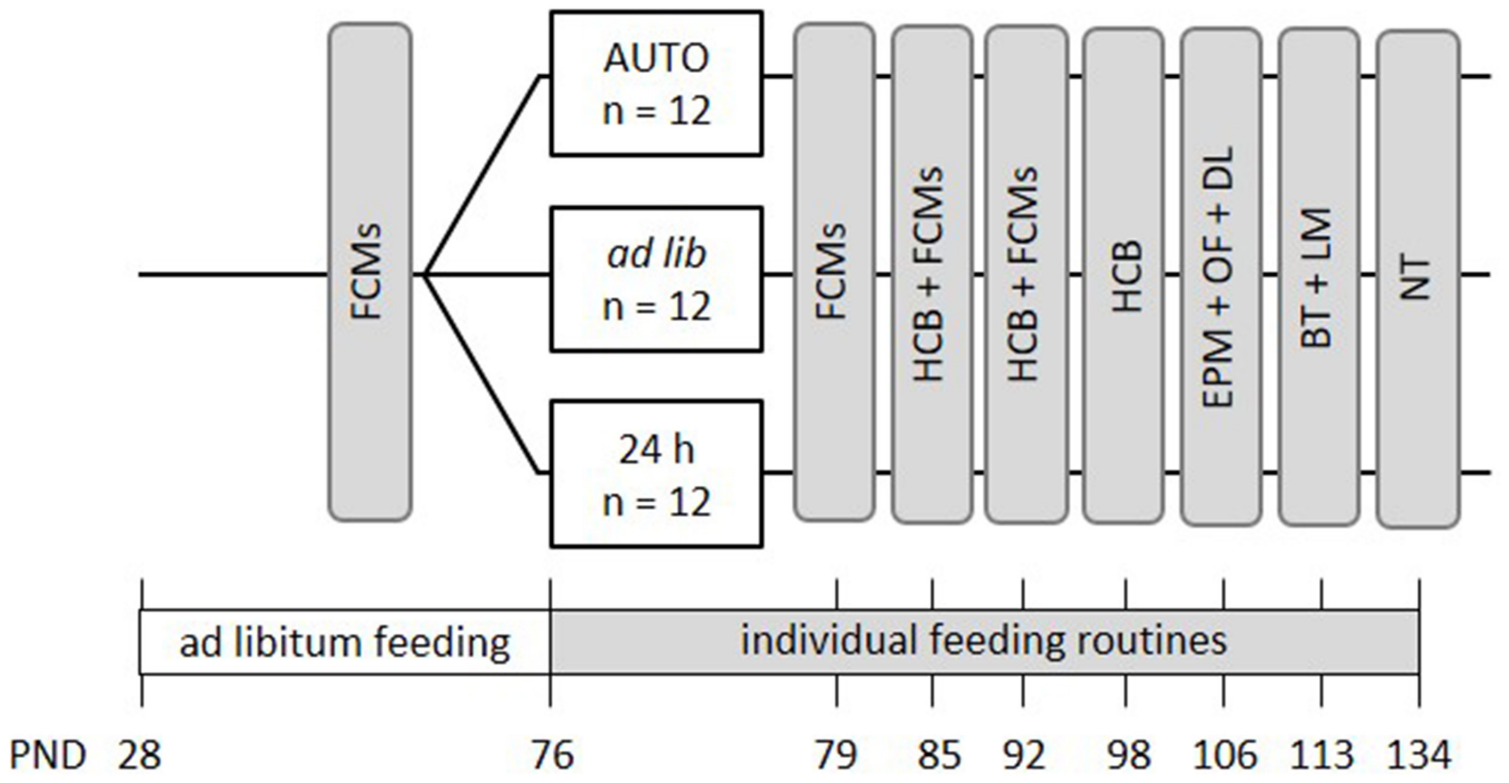
Schematic timeline of experiment 2. From PND 76 on, the animals were subjected to individual feeding routines and either fed *ad libitum* (*ad lib*), according to the 24 h schedule (24 h) or the automated feeding routine (AUTO). They were kept to this diet for a total experimental time of 9 weeks. Effects on animal welfare were evaluated by home cage behavior observations (HCB), measurements of fecal corticosterone metabolites (FCMs) and different behavioral tests, including the Elevated Plus Maze Test (EPM), the Open Field Test (OF), the Dark-Light Test (DL), the Barrier Test (BT), the Labyrinth-Maze Test (LM), and the Nest Test (NT). All information on PNDs have an accuracy of ±2 days.

Mice were kept to the different feeding routines for a total duration of 9 weeks (Figure 3). Similar to the first experiment, the effects of different feeding routines on welfare-related measures were evaluated over the course of this experimental phase. Concentrations of fecal corticosterone metabolites (FCMs) were assessed once before the onset of the different feeding routines and subsequently on a weekly basis during the first 3 weeks of the feeding paradigms (PND = 70-93). During the time from PND 82 to 99, basic activity levels and stereotypies in the home cage were recorded on one morning and one afternoon per week. Finally, a battery of behavioral tests was conducted between PNDs 106 and 134, including the Elevated Plus Maze Test (EPM), Open Field Test (OF), Dark Light Test (DL), Barrier Test (BT), Labyrinth-Maze Test (LM) and the Nest Test (NT) to investigate anxiety-like behavior and exploratory locomotion, spatial learning, and nesting behavior.

### Assessment of Welfare Indicators

In experiment 1, fecal corticosterone metabolites, home cage behavior, anxiety-like and exploratory behavior, as well as the voluntary interaction of the mice with the experimenter were assessed. Experiment 2 additionally tested spatial learning abilities and nesting behavior. Procedures for experiments 1 and 2 overlapped for the most part, but differed in some details (see section Experimental Design and Figures 1, 3 for more information).

#### Fecal Corticosterone Metabolites

Concentrations of fecal corticosterone metabolites (FCMs) were monitored non-invasively (30–32) to evaluate the activity of the hypothalamic-pituitary-adrenal (HPA) axis [for a review see (33)].

Directly after weighing, the mice were transferred to new Makrolon type III cages (“sample cages”), which were equipped with bedding material and enrichment as described under “Animals and housing conditions.” To receive comprehensive information on how different feeding routines affected the hormone levels over a whole day the animals were left in the sample cages for 24 h and then moved back to their respective home cages. Fecal samples were collected and frozen at −20°C until further preparation. For the determination of corticosterone metabolite concentration in the feces, the samples were dried and homogenized. Aliquots of 0.05 g were extracted with 1 ml of 80% methanol. Subsequently, a 5α-pregnane-3b,11b,21-triol-20-one enzyme immunoassay [established and validated by Touma et al. (31, 32)] was used for the analysis of corticosterone metabolites. EIA sensitivity was 1.7 ng/0.05 g and the intra- and inter-assay coefficients of variation were below 10 and 12%, respectively.

#### Home Cage Behavior

Spontaneous behavior in the home cage was recorded by live observations within the housing room under red light conditions after the onset of the experimental phase (Exp. 1: PNDs 76 – 93; Exp. 2: PNDs 82 – 99). Definitions of behaviors were based on previous publications (3, 29) (Table 1). Whether a mouse was active/inactive was recorded in both experiments to assess possible disturbances of the daily activity rhythm, which has been associated with a variety of health consequences (34–36). Stereotypic behaviors, which are repetitive and invariant behaviors without any obvious goal or function, were additionally included in experiment 2 (Table 1) as indicators of impaired welfare (37, 38). For data analysis, the percentage of intervals in which a mouse was active/inactive was calculated (39). The stereotypic behaviors were corrected for individual differences in activity. Daily observation sessions lasted 3 h and were divided into intervals of 15 s (Exp. 1) or 20 s (Exp. 2). The order in which the animals were observed was randomized but balanced across treatments. In experiment 1, the time from 2 to 5 p.m. was covered, a time at which all animals had had access to food for at least 2 h, resulting in similar satiation levels. Each mouse was observed 63 times, spread over nine observation sessions. In experiment 2, additionally the time from 10 a.m. to 1 p.m. was covered. Each mouse was observed 60 times for morning and afternoons each, spread over six observation sessions.

**Table 1:** Definitions of behaviors, based on previous publications (3, 29).

**Behavior**	**Definition**
Active/Inactive	The mouse is active when it shows any kind of motion. Tiny whisker, ear or tail movements are excluded. The mouse is inactive when it is was not active within one observation interval.
Stereotypies	The mouse shows stereotypies when it displays at least one of the following patterns three times or more within one observation interval: Patterned running: Running on the cage floor along fixed routes; Patterned climbing: Climbing at the cage lid along fixed routes; Circling lid: Climbing in tight circles on the cage lid.

*Active/inactive was determined in both experiments. Stereotypies were recorded only in experiment 2*.

### Behavioral Tests

All behavioral paradigms were performed during the animals' active phase when the lights were off in the housing room (between 1 and 6 p.m.), and mice were tested according to a randomized daily order, which was balanced across treatments. Barrier Test, Novel Cage Test, Interaction Test, and Nest Test were executed under red light conditions in the housing room and evaluated by live observations. For the tests on anxiety-like and exploratory behavior (Elevated Plus Maze Test, Dark Light Test, Open Field Test) and on spatial learning (Labyrinth-Maze Test) the animals were transferred to a separate testing room using a darkened transport box. All tests on anxiety-like and exploratory behaviors were recorded by a camera (Logitech Webcam Pro 9000) and automatically analyzed in real time by the video-tracking system ANY-maze [v. 4.75 (Exp. 1)/v. 5.33 (Exp. 2), Stoelting Co.,Wood Dale, USA], while the Labyrinth-Maze Test was manually evaluated by live observation. Between subjects, all test equipment was cleaned with 70% ethanol. Mice were given a pause of at least 48 h between individual tests.

#### Human-Animal Interaction Test

The Human-Animal Interaction Test was conducted in order to quantify the degree of voluntary interaction of the mice with the experimenter. The protocol was modified from Gouveia and Hurst (40). The test apparatus consisted of a standard Makrolon type III cage with clean bedding material. The experimenter placed one hand in the lower half of the cage, where it remained motionlessly during the test. The mouse was placed into a corner in the upper part of the cage and allowed to explore the area for 1 min. To assess the animal's willingness to interact with the experimenter, the latency to first enter the area around the hand, the number of entries to this area as well as the percentage of time spent in this area were measured.

#### Elevated Plus Maze Test

The Elevated Plus Maze Test evaluates anxiety-like and exploratory behavior [EPM; (41–43)]. This and similar tests are commonly used to diagnose welfare consequences of housing or treatment interventions (28, 44–48).

The wooden plus-formed apparatus with four arms (30 × 5 cm each) and a central square (5 × 5 cm) was elevated 50 cm above the floor. The setup was painted in a light gray and the arms were covered by a gray PVC inlay. Two opposing arms were enclosed by a wall of 20 cm height. The other two arms only had a small barrier of 0.4 cm to prevent the mice from falling off the apparatus. The illumination level was set to 25 lux in the center. After transportation to the testing room and 1 min in the transportation box, mice were placed on the apparatus with their head facing toward the closed arm of the apparatus pointing away from the experimenter. Immediately after starting the tracking software, the experimenter left the room. The mice were then allowed to explore the apparatus for 5 min. Measures that were used to examine the anxiety-like behavior were the time spent on the open arms compared to the total time spent on open and closed arms and the number of entries to the open arms compared to the total number of entries to open and closed arms. Exploratory behavior was assessed by comparing the total number of arm entries.

#### Dark Light Test

As further assessment of anxiety-like and exploratory behavior, the Dark Light Test (DL; Crawley and Goodwin (49) was executed. A standard Makrolon type III cage (37 × 21 × 15 cm) was modified to include a dark compartment, which made up one third of the cage. This area was separated by a black plastic panel from the rest of the cage, covered by a black plastic lid and the cage walls were darkened with black paint. Via a sliding door, the compartment was connected to the light compartment, which was not modified. The illumination level for the light compartment was set to 40 lux. For acclimatization, the mice were placed for 1 min in the dark compartment. Subsequently, the sliding door was opened, the ANY-maze tracking started and the experimenter left the room. The animals were then allowed to explore the apparatus for 5 min. The latency to the first entry to the light compartment and the time spent in the light compartment were used as assessment of the anxiety-like behavior. The number of entries to light compartment were used to evaluate the exploratory behavior.

#### Open Field Test

The Open Field Test [OF; Archer (50); Treit and Fundytus (51)] measures anxiety-like and exploratory behavior in an apparatus made of white coated plywood and consisted of a square arena (80 × 80 cm) surrounded by walls (42 cm). The illumination level in the center was set to be 35 lux. After 1 min in the transport box the mouse was placed into the apparatus with its head facing toward the lower left corner of the apparatus. The experimenter started the ANY-maze tracking and left the room immediately after letting go of the animal. The mouse then had 5 min to freely explore the apparatus. Measures for anxiety-like behavior were the duration spent in the center (defined as at least 20 cm distant from the wall) and the number of entries to the center. Exploratory behavior was assessed by the total distance traveled.

#### Novel Cage Test

This test investigated exploratory behavior in a new environment (44, 48, 52). Mice were placed into a standard Makrolon cage type III with a thin layer of bedding material and observed for 5 min. The number of rearings was recorded by live observation as a measurement of exploratory behavior.

#### Barrier Test

For evaluating the locomotive exploration, mice were individually placed into a standard Makrolon type III cage with a barrier of 3 cm height connecting the long sides of the cage in the middle (44, 46, 47). The mouse was placed into the right lower corner and the cage was covered with a transparent Plexiglas. The latency to the first crossing of the barrier within a maximum of 5 min was recorded by live observation.

#### Labyrinth-Maze Test

To test spatial learning abilities, the Labyrinth-Maze Test (LM) was performed. The apparatus consisted of a white platform (40 × 24 cm) with several transparent acrylic glass walls (15 cm). There were seven passageways in the walls to form a labyrinth. Only a restricted number led to the home cage, which was connected via a short tunnel (8 cm). The empty home cage was connected to the end of the LM while the animal was placed in an empty box protected from light for 1 min prior to testing. It was then placed on the start position of the LM, allowing it to freely explore the apparatus and find its way to the home cage for 5 min. Upon reaching the home cage, which acted as a reward, the mouse was given a 5 min pause. During this time, the LM was thoroughly cleaned with 70% ethanol and then the mouse was placed in the start position again to perform a second trial for 5 min maximum. The parameters measured were the time needed to exit the LM and the number of errors, meaning all transits through passageways that did not lead toward the exit.

#### Nest Test

Nesting behavior was assessed as an innate and highly motivated behavior in mice that has been shown to be influenced by various factors in housing procedures (53). Enrichment and old nesting material were removed from the home cage and each mouse received one cotton nestlet (5 × 5 cm, Zoonlab GmbH, Castrop-Rauxel, Germany) as nesting material. Cages were placed in racks and left undisturbed for the complete testing phase. Nesting performance was scored by two experienced researchers after 5 and 24 h using a scale adopted from Deacon (54) ranging from 1 (Nestlet to more than 90% intact) to 5 (>90% of the nestlet shredded, near perfect nest with walls higher than the mouse).

### Statistics

Graphs were created and the analysis of this study was conducted using the statistical software R [(55), Version 3.5.1], R Studio [(56), Version 1.1.453], and G^*^Power [(57), Version 3.1.9.4]. Data were analyzed using linear or linear mixed models. To test for normal distribution, residuals were examined graphically for homoscedasticity and outliers and additionally the Kolmogorov-Smirnov test and the Shapiro test were applied. If necessary, data were transformed using logarithmic or square root transformation (for detailed information, see Tables 2, 3).

**Table 2:** Statistics experiment 1: Presented are interaction and main effects of “time” and “feeding routine” (*F*-ratios, *p*-values, and estimated effect sizes) on weight, fecal corticosterone metabolite (FCM) concentrations, activity in the home cage and on individual common parameters that were assessed in the Human-Animal Interaction Test (HAI), the Elevated Plus Maze Test (EPM), the Dark-Light Test (DL), the Open Field Test (OF), and the Novel Cage Test (NC).

**Test**	**Parameter**	**Domain**	**Transf**.	**Feeding**			**Week**			**Interaction**		
				***F*-ratio**	***p*-value**	**η^2^ p**	***F*-ratio**	***p*-value**	**η^2^ p**	***F*-ratio**	***p*-value**	**η^2^ p**
Body weight [g]		Physiol.	–	16.930	**<0.001**	0.506	104.223	**<0.001**	0.760	68.017	**<0.001**	0.805
Corticosterone [ng/0.05 g]		Physiol.	lg	35.557	**<0.001**	0.683	3.307	**0.023**	0.091	0.941	0.469	0.054
Behavior	Active	Behav.	–	4.418	**0.020**	0.211						
HAI	Entries	hu./an. Interaction	–	0.506	0.608	0.030						
	Duration	hu./an. Interaction	–	1.524	0.233	0.085						
	Latency	hu./an. Interaction	lg	0.865	0.430	0.050						
EPM	Sum of arm entries (#)	Expl.	–	1.378	0.266	0.077						
	Relative open arm time	State anx.	–	6.397	**0.005**	0.279						
	Relative open arm entries	State anx.	–	2.698	**0.082**	0.141						
DL	Entries into light comp (#)	Expl.	–	6.965	**0.003**	0.297						
	Time in light comp (s)	State anx.	–	1.833	0.176	0.100						
	Latency to enter light comp (s)	State anx.	lg	0.836	0.444	0.048						
OF	Total distance (m)	Expl.	–	0.217	0.806	0.013						
	Center entries (#)	State anx.	–	1.545	0.228	0.086						
	Center time (s)	State anx.	sqrt	2.637	**0.087**	0.138						
NC	Rearings (#)	Expl.	–	0.837	0.442	0.048						

*Data were transformed (Transf.) whenever deviating from normal distribution. Lg, logarithmic; sqrt, square root. P-values in bold represent statistically significant differences (p < 0.05). State anx., state anxiety; expl., exploration; physiol., physiological; behav., behavioral; hu./an. Interaction, human-animal interaction*.

**Table 3:** Statistics experiment 2: Presented are interaction and main effects of “time”/“trial”/“daytime” and “feeding routine” (*F*-ratios, *p*-values, and estimated effect sizes) on weight, fecal corticosterone metabolite (FCM) concentrations, behavior in the home cage and on individual common parameters that were assessed in the Elevated Plus Maze Test (EPM), the Dark-Light Test (DL), the Open Field Test (OF), the Barrier Cage Test (BT), the Labyrinth-Maze Test (LM), and the Nest Test (NT).

**Test**	**Parameter**	**Domain**	**Transf**.	**Feeding**			**Week / Trial / Daytime**			**Interaction**		
				***F*-ratio**	***p*-value**	**η^2^ p**	***F*-ratio**	***p*-value**	**η^2^ p**	***F*-ratio**	***p*-value**	**η^2^ p**
Body weight [g]		Physiol.	–	29.671	**<0.001**	0.650	41.181	**<0.001**	0.555	33.065	**<0.001**	0.667
Corticosterone [ng/0.05 g]		Physiol.	lg	33.762	**<0.001**	0.672	1.817	**0.015**	0.052	11.837	**<0.001**	0.418
Behavior	Active	Behav.	–	11.314	**<0.001**	0.407	75.461	**<0.001**	0.696	36.269	**<0.001**	0.687
EPM	Sum of arm entries (#)	Expl.	–	4.080	**0.026**	0.198						
	Relative open arm time	State anx.	–	1.609	0.215	0.089						
	Relative open arm entries	State anx.	–	0.804	0.456	0.046						
DL	Entries into light comp. (#)	Expl.	–	3.535	**0.041**	0.176						
	Time in light comp. (s)	State anx.	–	0.159	0.854	0.010						
	Latency to enter light comp. (s)	State anx.	lg	5.418	**0.009**	0.247						
OF	Total distance (m)	Expl.	–	2.683	0.084	0.140						
	Center entries (#)	State anx.	–	1.068	0.355	0.061						
	Center time (s)	State anx.	sqrt	2.061	0.144	0.111						
BT	Latency (s)	Expl.	lg	0.309	0.736	0.018						
LM	Errors made (#)	Learning	lg	0.215	0.807	0.007	35.675	**<0.001**	0.358	0.093	0.912	0.003
	Time needed (s)	Learning	lg	0.626	0.538	0.019	51.167	**<0.001**	0.440	0.581	0.562	0.018
Nest test	Score	Nesting	–	6.283	**0.005**	0.276	31.931	**<0.001**	0.492	0.863	0.431	0.050

*Data were transformed (Transf.) whenever deviating from normal distribution. Lg, logarithmic; sqrt, square root. P-values in bold represent statistically significant differences (p < 0.05). State anx. = state anxiety; expl. = exploration; physiol. = physiological; behav. = behavioral*.

Body weight, fecal corticosterone metabolite concentrations, home cage behavior (experiment 2) and nesting behavior were analyzed using a repeated measures analysis of variance (ANOVA) with “feeding routine” as fixed between subjects factors, “week”/“daytime”/“trial” as fixed within subjects factor and individuals as random factors. Behavioral data were analyzed using a Univariate ANOVA with “feeding routine” as fixed between-subjects factor. In experiment 2, all procedures were executed in two separate batches. Therefore, batches were always included as random factor, to control for possible differences not caused by experimental procedures. In case of significant main or interaction effects, Bonferroni-Holm *post hoc* comparisons were conducted. Partial eta squared (η^2^p) was calculated as a measure of the magnitude of the reported effects (58). Stereotypic behavior was analyzed using the non-parametric Kruskal-Wallis test. For *post hoc* pairwise comparisons Mann-Whitney-*U*-tests with Bonferroni-Holm correction were conducted. Differences were considered to be significant at *p* < 0.05.

According to a statistical power analysis, biologically relevant differences could be detected with a power of 80% for medium effect sizes >0.32 (repeated measures ANOVAs) or large effect sizes >0.54 [univariate ANOVAs, (59)].

## Results

### Experiment 1

#### Validation of the Alternative Feeding Routines

In experiment 1 *ad libitum* feeding was compared to a 24 h schedule and a 4 h removal group.

**Figure 4:**
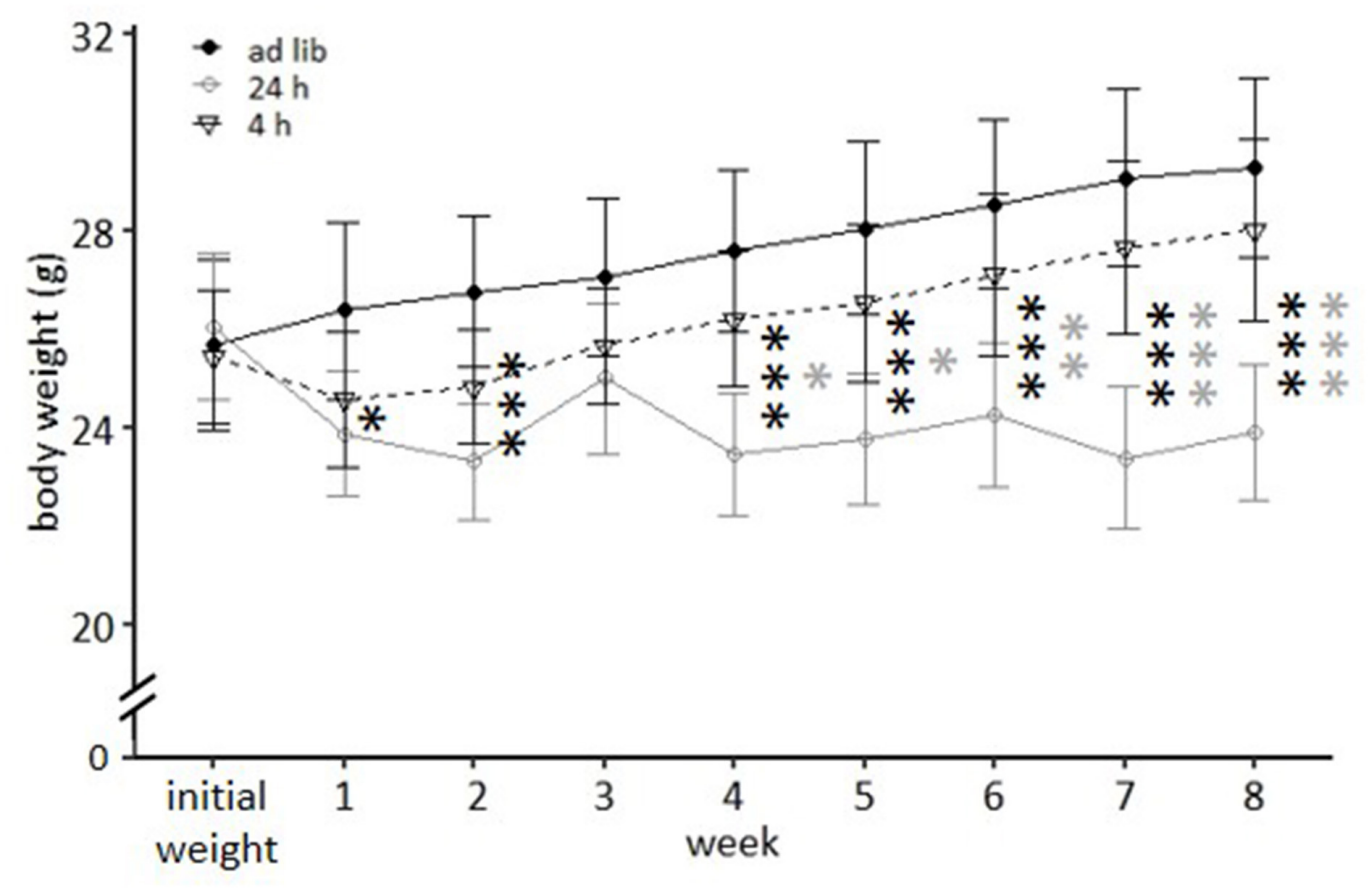
Weight development under different feeding routines over a period of 8 weeks. Initial weight, *Ad libitum* feeding for all groups, before onset of feeding routines; *Ad lib, Ad libitum* group; 24 h, 24 h schedule group; 4 h, 4 h removal group. Data are presented as means ± SD. Statistics: repeated measures ANOVA, *post hoc*: Bonferroni-Holm corrected; sample size: *n* = 12 per group; ^*^p < 0.05, ^**^p < 0.01, ^***^p < 0.001. Gray asterisks: comparison 24 and 4 h, black asterisks: comparison *ad lib* and 24 h.

The repeated measures ANOVA revealed a significant main effect of feeding routine [*F*_(2, 33)_ = 16.930, *p* < 0.001] and week on body weight [*F*_(8, 264)_ = 104.223, *p* < 0.001], as well as a significant interaction between week and feeding routine [*F*_(16, 264)_ = 68.017, *p* < 0.001; Figure 4].

Mice of the *ad libitum* group and the 4 h removal group continuously gained weight during the experimental phase and according to *post hoc* analysis did not differ significantly from each other (*p*-value range: 0.468–1). Animals of the 24 h schedule were successfully maintained at 90–95% of their initial body weight and differed therefore significantly from *ad libitum* fed mice throughout the experimental phase (week 1: *p* = 0.047; weeks 2, 4–8: *p* < 0.001), except during week 3 (*p* = 0.319). Significant differences existed also between mice of the 24 h schedule and the 4 h removal after week 3 (week 4 and 5: *p*-value range 0.016–0.014; weeks 6–8: *p* < 0.001).

#### Effects of Feeding Routines on Welfare Indicators

##### Fecal corticosterone metabolites

**Figure 5:**
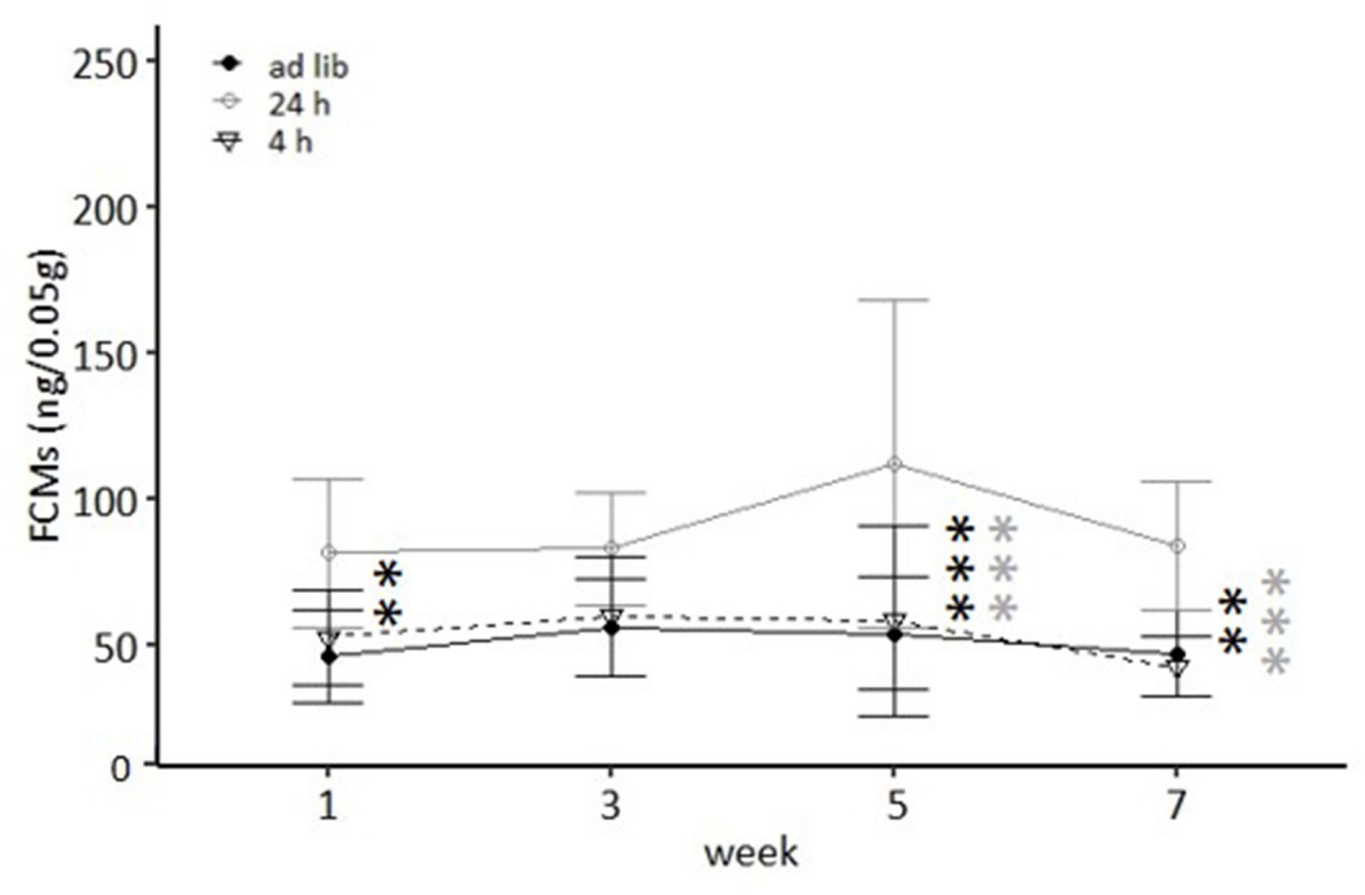
Levels of fecal corticosterone metabolites (FCMs). *Ad lib, Ad libitum* group; 24 h, 24 h schedule group; 4 h, 4 h removal group. Data presented as means ± SD. Statistics, repeated measures ANOVA; *Post hoc*, Bonferroni-Holm corrected; sample size: *n* = 12 per group; ^**^p < 0.01, ^***^p < 0.001. Gray asterisks: comparison 24 and 4 h, black asterisks: comparison *ad lib* and 24 h.

Both week and feeding routines had a significant effect on concentrations of fecal corticosterone metabolites (FCMs) in the repeated measures ANOVA [Feeding routines: *F*_(2, 33)_ = 35.557, *p* < 0.001; Week: *F*_(3, 99)_ = 3.307, *p* = 0.023; Table 2]. In the *post hoc* analysis no differences in concentrations between the *ad libitum* group and the 4 h removal group were found for any measurement (*p* = 1 for all comparisons; Figure 5). In contrast, mice of the 24 h schedule showed significantly higher concentrations compared to mice of the *ad libitum* group in week 1 (*p* = 0.002), week 5 (*p* < 0.001), and week 7 (*p* = 0.001) as well as compared to animals of the 4 h group in week 5 and 7 (*p* < 0.001 for both comparisons).

##### Home cage behavior

The univariate ANOVA revealed a significant main effect of feeding routines on activity levels in the home cage [*F*_(2, 33)_ = 4.418, *p* = 0.020; Table 2]. *Post hoc* analysis indicated that mice of the 4 h removal group were less active than animals of the 24 h schedule group (*p* = 0.017). There were no differences between *ad libitum* fed mice and mice of the 24 h schedule or the 4 h removal (*p* = 0.226 for both comparisons).

##### Behavioral tests

**Figure 6:**
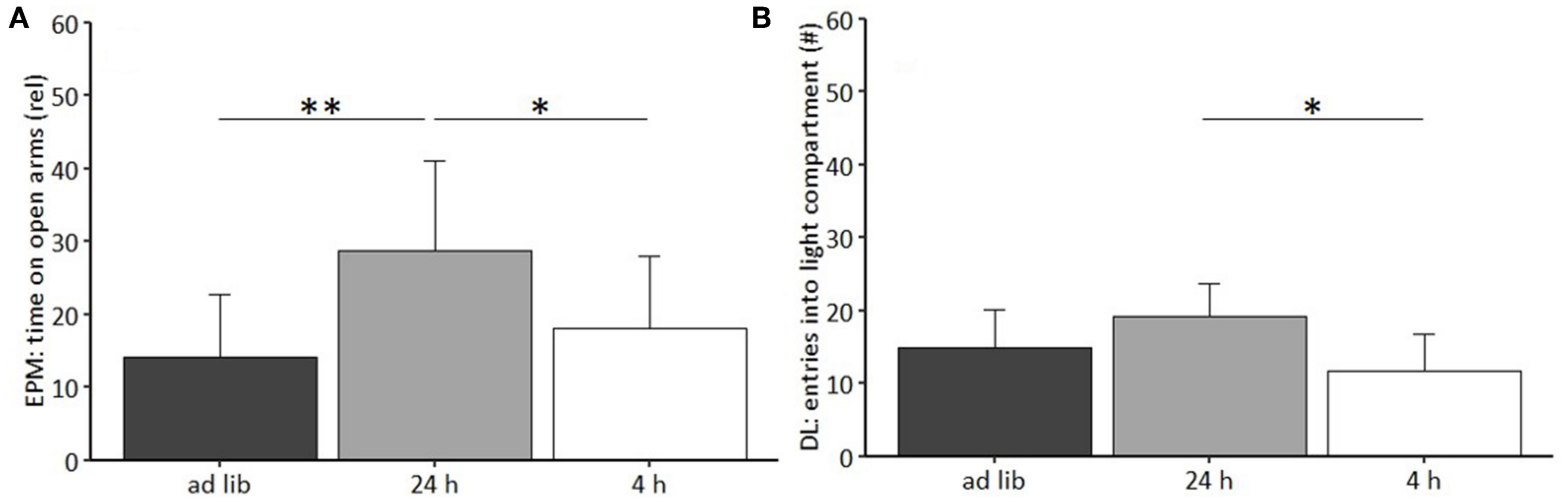
Anxiety-like and exploratory behavior. **(A)** Elevated Plus Maze (EPM): Relative time on open arms; **(B)** Dark Light (DL): Number of entries to light compartment. *Ad lib, Ad libitum* group; 24 h, 24 h schedule group; 4 h, 4 h removal group. Data are presented as means ± SD. Statistics: ANOVA, *post hoc*: Bonferroni-Holm corrected; sample size: *n* = 12 per group; ^*^p < 0.05, ^**^p < 0.01.

Regarding anxiety-like and exploratory behavior, the univariate ANOVA uncovered a significant effect of feeding routine on the time spent on the open arms of the Elevated Plus Maze Test [EPM, *F*_(2, 33)_ = 6.397, *p* = 0.005; Figure 6A) and the number of entries to the light compartment of the Dark Light Test [DL, *F*_(2, 33)_ = 6.965, *p* = 0.003, Figure 6B). *Post hoc* analysis indicated lower levels of anxiety-like behavior for mice of the 24 h schedule group, which spent more time on the open arms than animals of both other groups (*Ad lib*: *p* = 0.005, 4 h removal: *p* = 0.033). Furthermore, exploratory behavior was lower for animals of the 4 h removal group than for animals of the 24 h schedule, as indicated by a lower number of entries to the light compartment of the DL (*Post hoc, p* = 0.002).

No significant main effects were found for the other parameters of the EPM (sum of arm entries, relative number of open arm entries) and the DL (latency to enter and time in the light compartment). Furthermore, no significant main effects were revealed for the Human-Animal Interaction Test (HAI), the Novel Cage Test (NC), and the Open Field Test (OF; for statistical details see Table 3).

#### Summary

A continuous weight gain was shown for mice of the *ad libitum* group and the 4 h removal group, while animals of the 24 h schedule group where kept at the target weight. Overall, there was little evidence for distinct effects of feeding routines on animal welfare indicators. However, there was some indication for lower activity and exploratory locomotion in the 4 h removal group as well as lower anxiety-like behavior and higher FCM concentrations in the 24 h schedule group.

### Experiment 2

#### Validation of the Alternative Feeding Routines

In the second experiment, *ad libitum* feeding was compared to the 24 h schedule and the automated feeding routine (AUTO) group, in which six small food pellets were delivered by an automated apparatus throughout the day. There was a significant main effect of feeding routine [*F*_(2, 32)_ = 29.671, *p* < 0.001] and week [*F*_(9, 297)_ = 41.181, *p* < 0.001] on body weights, as well as a significant interaction between week and feeding routine [*F*_(18, 297)_ = 33.065, *p* < 0.001; Table 3].

**Figure 7:**
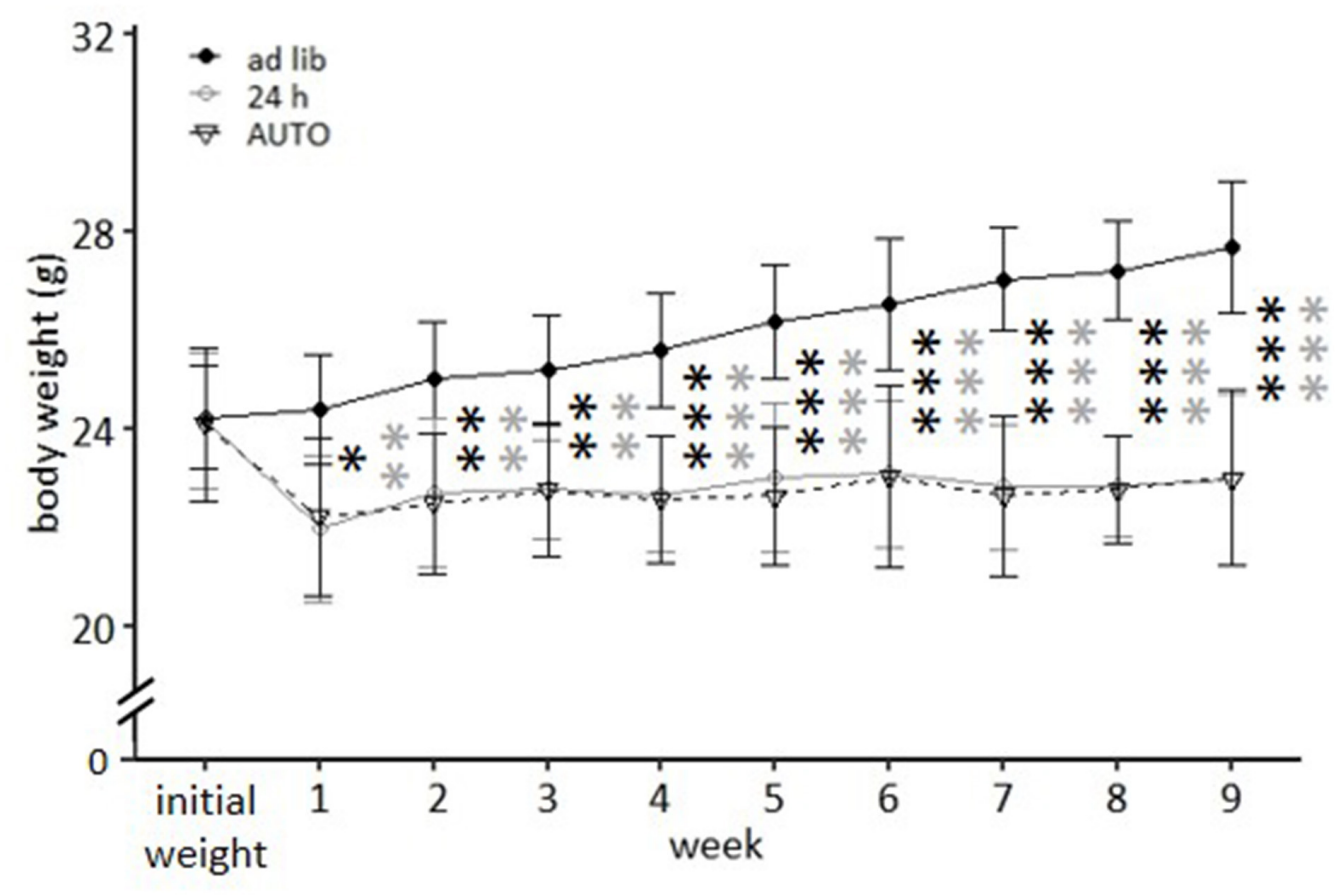
Weight development under different feeding routines over a period of 9 weeks. Initial weight, *Ad libitum* feeding for all groups, before onset of feeding routines, *Ad lib, Ad libitum* group; 24 h, 24 h schedule group; AUTO, Automated feeding routine group. Data are presented as means ± SD. Statistics: repeated measures ANOVA, *post hoc*: Bonferroni-Holm corrected; sample size: *n* = 12 per group; ^*^p < 0.05, ^**^p < 0.01, ^***^p < 0.001. Gray asterisks: comparison *ad lib* and 24 h, black asterisks: comparison *ad lib* and AUTO.

While *ad libitum* fed mice continuously gained weight, mice of the 24 h schedule and the AUTO group were successfully maintained at 90–95% of their *ad libitum* weight during the experimental phase. This is reflected in the *post hoc* analysis, which indicated significant differences when comparing *ad libitum* fed mice to either mice of the 24 h schedule or of the AUTO group from week 1 on (week 1–3: *p*-value range: 0.001–0.014; week 4–9: *p* ≤ 0.001 for all comparisons, Figure 7).

#### Effects of Feeding Routines on Welfare Indicators

##### Fecal corticosterone metabolites

**Figure 8:**
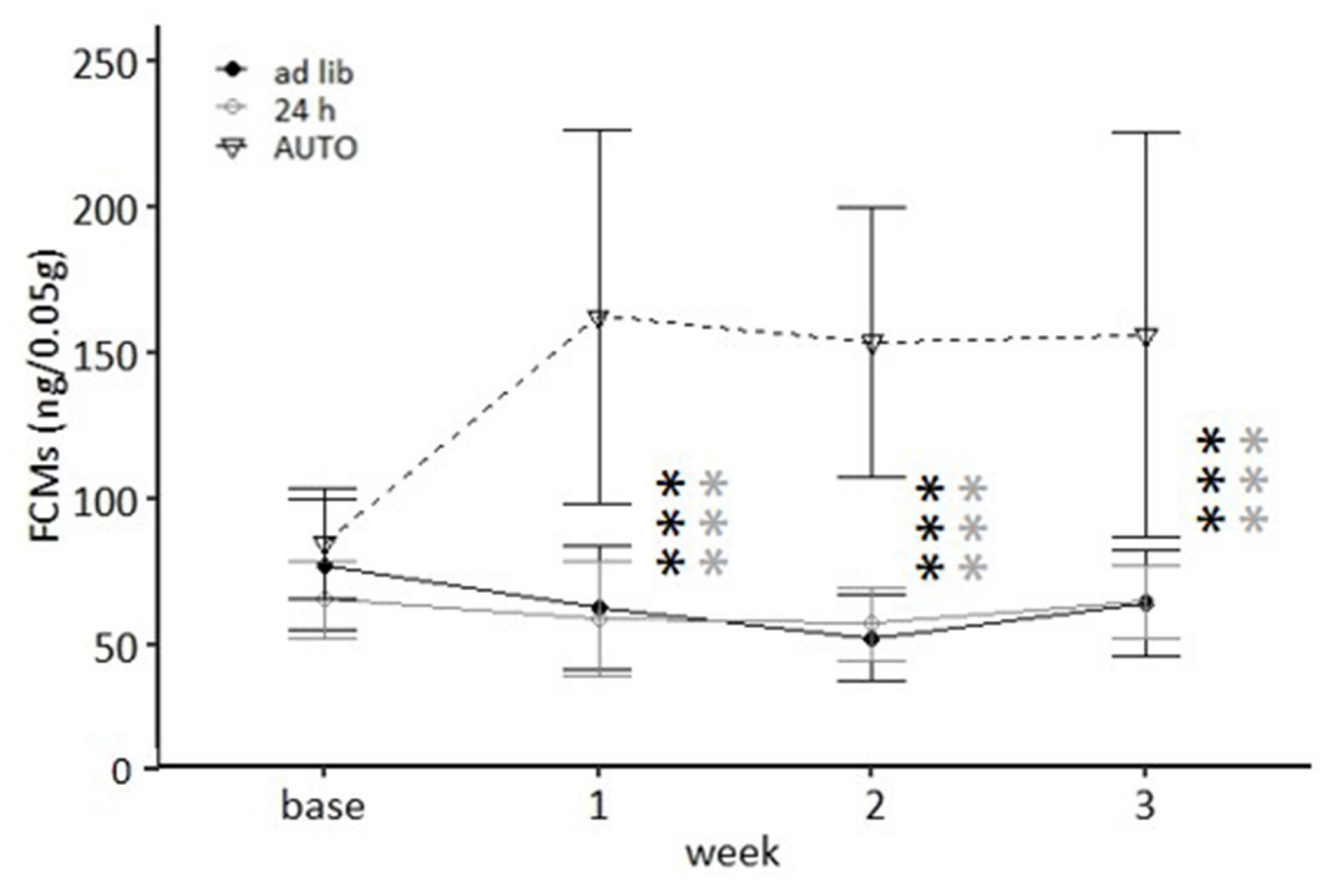
Levels of fecal corticosterone metabolites (FCMs). Base, Baseline; *ad libitum* feeding for all groups. *Ad lib, Ad libitum* group; 24 h, 24 h schedule group; AUTO, Automated feeding routine group. Data presented as means ± SD. Statistics, repeated measures ANOVA; *Post hoc*, Bonferroni-Holm corrected); sample size: *n* = 12 per group; ^***^p < 0.001. Gray asterisks: comparison *ad lib* and 24 h, black asterisks: comparison *ad lib* and AUTO.

Feeding routine [*F*_(2, 33)_ = 33.762, *p* < 0.001] as well as the week by feeding routine interaction [*F*_(6, 99)_ = 11.837, *p* < 0.001; Table 3] had a significant effect on corticosterone metabolite concentrations. *Post hoc* analysis indicated no differences between the *ad libitum* group and the 24 h schedule group before or after the start of the experimental phase (*p* = 1 for all comparisons; Figure 8). Mice of the AUTO group showed significantly higher FCM concentrations from the first week of the alternative feeding routines on, compared to both of the other groups (week 1–3: *p* < 0.001 for all comparisons).

##### Home cage behavior

**Figure 9:**
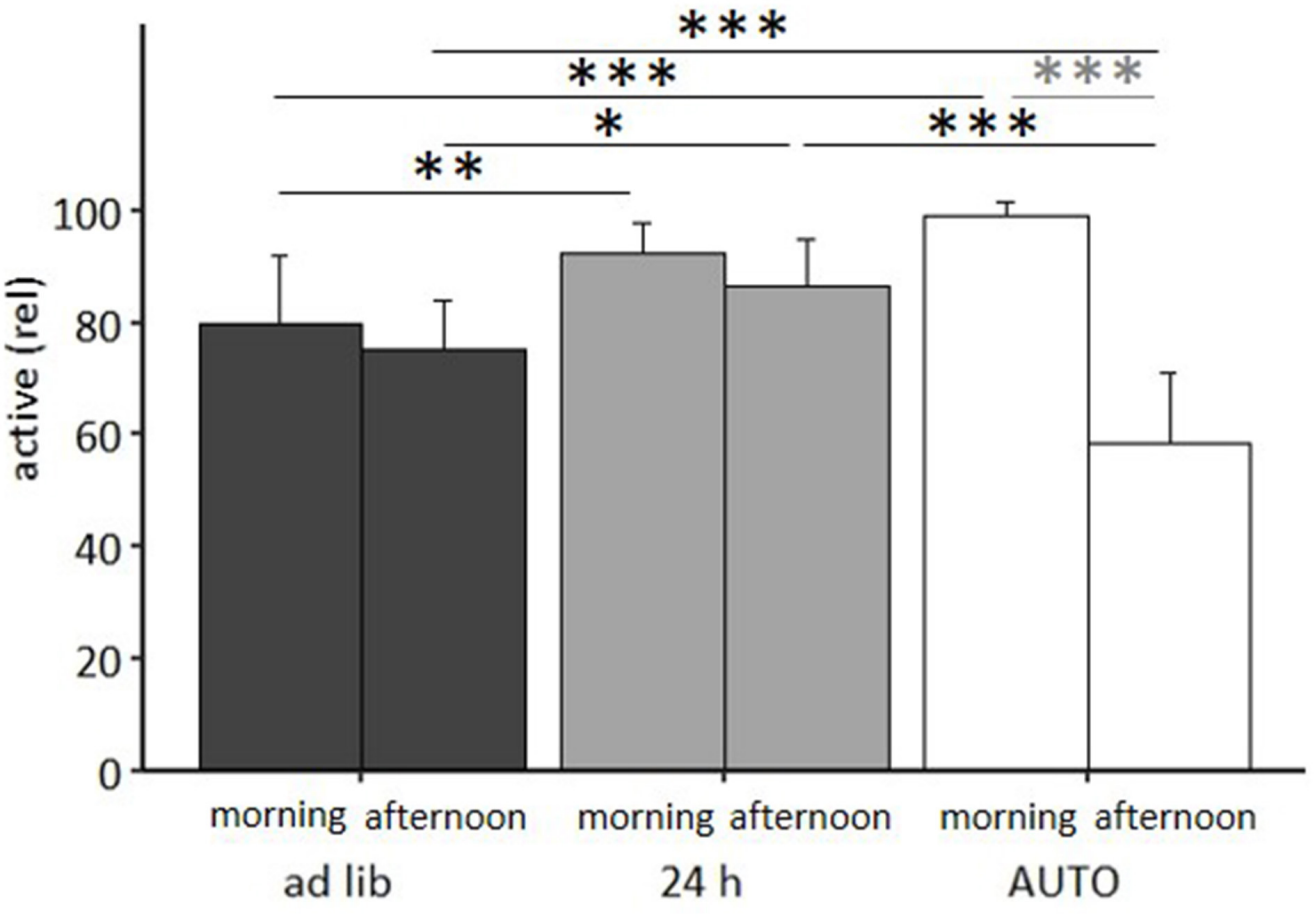
Activity in the home cage. *Ad lib, Ad libitum* group; 24 h, 24 h schedule group; AUTO, Automated feeding routine group. Data presented as mean ± SD. Statistics: mixed-model ANOVA, *Post hoc*: Bonferroni-Holm corrected; sample size: *n* = 12 per group; ^*^p < 0.05, ^**^p < 0.01, ^***^p < 0.001.

The mixed model ANOVA with repeated measures revealed a significant main effect of feeding routine [*F*_(2, 33)_ = 11.314, *p* < 0.001] and daytime [*F*_(1, 33)_ = 75.461, *p* < 0.001] as well as a significant feeding routine by daytime interaction on activity [*F*_(2, 33)_ = 36.269, *p* < 0.001, for statistical details, see Table 3]. According to *post hoc* analysis, animals of the 24 h schedule group were generally more active than animals of the *ad libitum* group, as seen by higher activity levels during mornings (*p* = 0.009) and afternoons (*p* = 0.011). A comparison of activity during mornings and afternoons within the groups resulted in a significant difference only for the AUTO group (*p* < 0.001, Figure 9). Correspondingly, the AUTO group showed higher activity levels than *ad libitum* fed animals during mornings (*p* < 0.001) and lower levels during afternoons compared to both of the other groups (*Ad lib*: *p* < 0.001, AUTO: *p* < 0.001).

There was a significant main effect of feeding routines on the frequencies of stereotypies (Kruskal-Wallis test, *H* = 6.606, *p* = 0.037; Table 4). Pairwise comparisons using the Mann-Whitney-*U*-test with Bonferroni-Holm correction indicated that animals of the AUTO group showed significantly more stereotypic behavior than animals of the *ad libitum* group (*U* = 33, *p* = 0.039). No differences were found between the *ad lib* group and the 24 h schedule group (*U* = 59, *p* = 0. 343) nor between the 24 h schedule group and the AUTO group (*U* = 48, *p* = 0.294).

**Table 4:** Relative frequencies of stereotypic behaviors (interval with stereotypies/active intervals).

**Group**	**Median**	**1. Quartile**	**3. Quartile**	**Max**	**Min**
*Ad lib*	0	0	0	1.98	0
24 h	0	0	1.17	4.04	0
AUTO	1.16	0	2.39	22.86	0

*Presented are median, 1. and 3. quartile as well as the maximum and minimum frequencies per day and group*.

##### Behavioral tests

**Figure 10:**
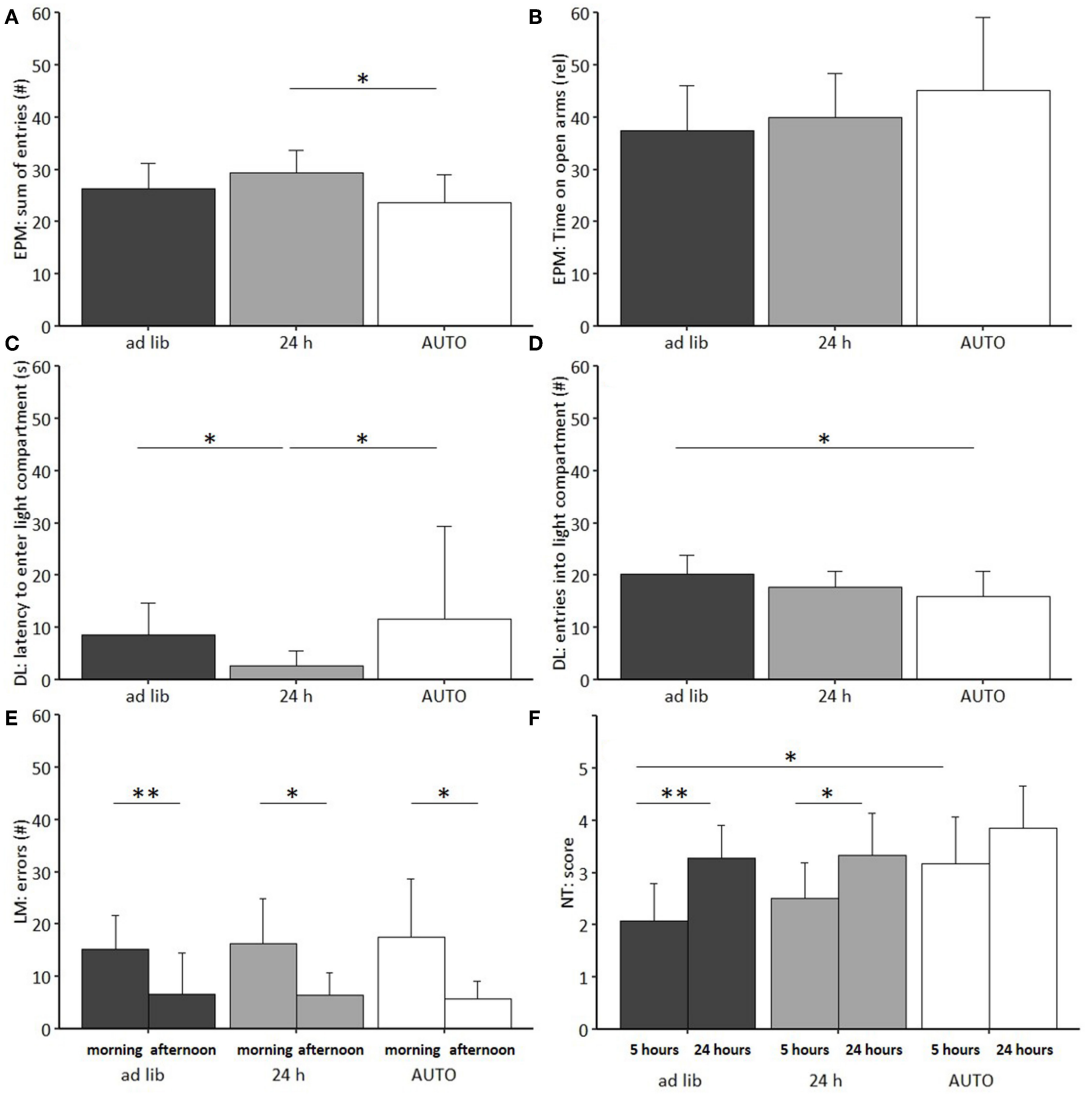
Behavioral tests on anxiety-like and exploratory behavior, spatial learning and nesting behavior. **(A)** Elevated Plus Maze (EPM): Sum of entries into open and closed arms, **(B)** EPM: Relative time on open arms, **(C)** Dark Light (DL): Latency to first enter the light compartment, **(D)** DL: Number of entries to light compartment, **(E)** Labyrinth-Maze Test (LM): Number of errors per trial. **(F)** Nest Test (NT): Score after 5 and 24 h. *Ad lib, Ad libitum* group, 24 h, 24 h schedule group; AUTO, Automated feeding routine group. Data presented as mean ± SD. Statistics: Repeated measures ANOVA, *post hoc*: Bonferroni-Holm corrected; sample size: *n* = 12 per group; ^*^p < 0.05, ^**^p < 0.01.

Regarding anxiety-like and exploratory behavior, a significant main effect of feeding routine was found for the number of arm entries on the EPM [ANOVA; *F*_(2, 33)_ = 4.08, *p* = 0.026], the number of entries to the light compartment [ANOVA; *F*_(2, 33)_ = 3.535, *p* = 0.040], as well as on the latency to enter the light compartment of the DL [*F*_(2, 33)_ = 5.418, *p* = 0.009; Figures 10A–D].

*Post hoc* analysis revealed lower levels of anxiety-like behavior for animals of the 24 h schedule group than for animals of the *ad libitum* or the AUTO group, as indicated by the shortest latency to enter the light compartment of the DL (*Ad lib*: *p* = 0.014, AUTO: *p* = 0.027). Exploration was lowest for the AUTO group, indicated by a lower number of entries to the light compartment in the DL compared to *ad libitum* fed mice (*p* = 0.037) and a lower number of arm entries in the EPM compared to animals of the 24 h schedule group (*p* = 0.022). No significant main effects were found either in the Open Field Test (OF) or in the Barrier Test (BT).

The repeated measures ANOVA in the Labyrinth-Maze Test (LM) indicated a significant main effect of trial for both errors made [*F*_(1, 64)_ = 35.675, *p* < 0.001] and time needed [*F*_(1, 65)_ = 51.167, *p* < 0.001]. *Post hoc* analysis confirmed that all groups reduced the number of errors (*Ad lib*: *p* = 0.004, 24 h: *p* = 0.020, AUTO: *p* = 0.015, Figure 10F) and the latency to finish the maze (*p* < 0.001 for all comparisons) from trial one to trial two, indicating a successful learning process. No differences were found between groups (Table 3).

Feeding routine [Repeated measures ANOVA; *F*_(2, 33)_ = 6.283, *p* = 0.005] and time [*F*_(1, 33)_ = 31.931, *p* < 0.001; Figure 10E; Table 3] had a significant main effect on nesting behavior. *Post hoc* analysis indicated a significant increase of the nesting scores from 5 to 24 h within the *ad libitum* and the 24 h schedule group (*Ad lib*: *p* = 0.002, 24 h: *p* = 0.050), but not in the AUTO group (*p* = 0.164). Additionally, the AUTO group had a significantly higher score after 5 h than the *ad libitum* group (*p* = 0.010).

#### Summary

In the second experiment, *ad libitum* fed mice showed a continuous weight gain, while animals of both of the other feeding routine groups were successfully kept at a lower body weight. With respect to most welfare indicators, no significant treatment differences were detected. However, there was some indication for lower levels of anxiety-like behavior and higher levels of activity in the 24 h schedule compared to the *ad libitum* group. In contrast, higher levels of corticosterone metabolites and higher frequencies of stereotypic behaviors were found for the AUTO group.

## Discussion

The negative effects of *ad libitum* feeding and the resulting overweight on the health of laboratory mice are well-established [reviewed by (60)]. Nonetheless, it is still the standard procedure in laboratories around the globe. The most widespread alternative, the 24 h schedule, consists of feeding a limited amount of food once per day and thereby reducing the animals' body weight to a predefined value. From a health perspective, this feeding routine has repeatedly been linked to positive effects, including for example an increased life span or a decreased disease susceptibility [reviewed by (19–22)]. Previous studies suggest that the observed health benefits most likely result from the decrease in body weight rather than the change in food delivery [e.g., (23, 24)], emphasizing the role of weight reduction for refining standard feeding routines. Despite this evidence, however, the repeated removal of food for up to 24 h is classified as “mildly severe” according to the current European Directive 2010/63/EU^1^, suggesting a negative effect on health and welfare at least in comparison to the non-classified standard *ad libitum* feeding method.

Against this background, the present study aimed at systematically investigating the effects of three different feeding routines compared to the wildly adopted “norm” of *ad libitum* feeding. In particular, a 24 h schedule group, an AUTO group, characterized by an automated supply of small pieces of food across the day, and a 4h removal group, characterized by daily removal of food for 4h, were evaluated regarding their effects on several established welfare indicators [for a review see (61)]. Additionally, it was studied, whether the removal of food for 4 h daily in a period of high feeding activity (see Figure 2A), was sufficient to downregulate body weights in the same way as it was achieved by feeding the animals once (24 h schedule) or several times per day (AUTO group) a reduced amount of food.

Removing the food for 4 h per day, however, did not lead to an overall reduction of body weights, indicating that this feeding routine is not suited to counteract the negative effects of overfeeding. Furthermore, no significant impairment or improvement of the animals' welfare was found compared to the *ad libitum* group, questioning the overall usefulness of this method for refining standard feeding routines under laboratory conditions in the long term.

In contrast, animals of the AUTO group were successfully kept at lower body weights. Concerning welfare indicators, the most distinct differences were found with respect to fecal corticosterone metabolites (FCMs) and stereotypic behaviors. In fact, the three-fold increased concentrations of FCMs found in the present study are a good indication for an activation of the HPA axis, which represents a main stress system in mammals [for a review see (30–33, 62)]. Other factors known to cause such an effect are severe stressors, such as intra-bone marrow transplantations (63), but also less severe stressors, e.g., increased housing density or social defeat (64, 65).

Additionally, higher levels of stereotypic behaviors were found in the AUTO group compared to the *ad libitum* group. Stereotypies are repetitive, invariant behavioral patterns, which lack any obvious goal or function (37, 38). Possible causes for the development include the frustration of not being able to perform highly motivated behaviors, the inability to cope with the environment or a dysfunction of the central nervous system (66). Furthermore, they are known to predominantly develop in barren and small cages (3, 67–69), and are thus considered to be indicative of impaired welfare (37, 38).

Further significant differences were found between the home cage activity pattern of the AUTO group and the activity pattern of the *ad libitum* and the 24 h schedule group. Although a change of the sleeping behavior has been discussed in the context of compromised welfare in rats (70), the observed change in activity pattern of the AUTO group is more likely caused by an adjustment to the delivery time points of the food pellets. Such a phenomenon is known as food entrainment, which is often accompanied by anticipatory activity [e.g., (71–73)]. Likewise, higher activity levels of the AUTO group during mornings could also have caused higher scorings in the Nest Test after 5 h. Furthermore, the low activity levels during the afternoon coincided with the time point of behavioral testing, possibly explaining why animals of the AUTO group were characterized by lower levels of exploratory behavior in two out of four tests. Overall, considering the higher levels of FCMs and of stereotypic behaviors, the automated feeding does not represent a suitable long-term feeding routine.

Concerning the 24 h schedule, hardly any differences in welfare indicators in comparison to the *ad libitum* group were found. Solely the time spent on the open arms of the EPM apparatus was significantly increased (Exp. 1) and the latency to enter the light compartment of the DL apparatus significantly decreased (Exp. 2), pointing toward lower levels of anxiety-like behavior in the 24 h schedule mice in comparison to the *ad libitum* mice. With respect to the FCMs, however, higher levels of corticosterone metabolite concentrations were found in experiment 1, which might be considered indicative of higher stress levels at first glance. However, since no baseline values were assessed in the first experiment, it cannot be excluded that these differences existed already before the onset of the different feeding routines. Furthermore, although the levels of corticosterone metabolite concentrations of the 24 h group in experiment 1 were significantly higher than in the *ad libitum* group, they were still considerably lower than in the AUTO group with a difference of about 45%.

Considering the overall picture, the 24 h schedule reliably reduced the animals' body weights and did not cause distinct negative effects on welfare. With respect to the EU guidelines and in particular in comparison to *ad libitum* feeding, this might question the classification of the 24 h schedule as “mildly severe.” Before drawing general conclusions, however, females and other strains should be investigated [e.g., (74, 75)].

Besides the already widespread use for motivational purposes [e.g., (13–18)], the 24 h schedule may thus be seen as a good candidate for a long-term feeding routine. Nonetheless, from a practical point of view, the 24 h schedule, as it was conducted here, requires daily weighing and close monitoring of the animals, thereby increasing the workload compared to *ad libitum* feeding. Additionally, in pair-housed or group-housed animals, the allocation of food to the individual animals requires additional effort.

Yet, solutions that would make this possible even in group-housed mice already exist by marking mice individually with RFID transponders and giving them selective access to feeders. Arguably, this solution is not yet applicable on a large scale, as it is quite complex. However, in the near future, technical developments are very likely to provide the means for applying a 24 h schedule as a general housing routine.

An alternative approach to preventing the negative effects of *ad libitum* feeding could be to use a diet with a lower energy content. Further studies are needed to examine if existing low-energy diets meet the nutritional demands of laboratory mice and test whether such a diet keeps the body weights low at the long-term.

Today, *ad libitum* feeding is widely accepted as the standard feeding routine for the housing of laboratory rodents. However, detrimental effects on health are well-established. In line with this, the present study shows that alternative feeding routines might improve the animals' health without negatively affecting other welfare parameters. From a broader perspective, these results emphasize that theoretical considerations or subjective evaluations cannot be considered sufficient for the classification of experimental or housing routines. Instead, systematic and evidence-based severity assessments are needed to evaluate any procedure involving living animals [e.g., (27, 28)].

## Data Availability Statement

The datasets generated for this study are available on request to the corresponding author.

## Ethics Statement

All procedures complied with the regulations covering animal experimentation within Germany (Animal Welfare Act) and the EU (European Communities Council DIRECTIVE 2010/63/EU)^1^ and were approved by the local (Gesundheits und Veterinäramt Münster, Nordrhein-Westfalen) and federal authorities (Landesamt für Natur, Umwelt und Verbraucherschutz Nordrhein-Westfalen “LANUV NRW”).

## Author Contributions

SR, NS, and SK conceived the study. JF-D, NS, SR, SK, and VK designed the experiments. JF-D, LB, and LS performed the experiments, while JF-D, SR, and VK supervised the project. RP determined the hormonal data. JF-D analyzed the data and wrote the initial draft of the manuscript. LB, LS, NS, RP, SR, SK, and VK revised it critically for important intellectual content.

### Conflict of Interest

The authors declare that the research was conducted in the absence of any commercial or financial relationships that could be construed as a potential conflict of interest.

## Footnotes

^1^Directive 2010/63/EU of the European Parliament and of the Council of 22 September 2010 on the protection of animals used for scientific purposes. Off J Eur Union 276:33-79.

## References

[1] Federal Ministry of Food and Agriculture (BMEL) Number of Experimental Animals. (2017). Available online at: https://www.bmel.de/DE/Tier/Tierschutz/_texte/Versuchstierzahlen2017.html (accessed May 13, 2019).

[2] Russell WMS Burch RL The Principles of Humane Experimental Technique, Vol. 238 London: Methuen (1959).

[3] Gross AN Richter SH Engel AKJ Würbel H. Cage-induced stereotypies, perseveration and the effects of environmental enrichment in laboratory mice. Behav Brain Res. (2012) 234:61–8. 10.1016/j.bbr.2012.06.007 22721674

[4] Freymann J Tsai P-P Stelzer H Hackbarth H The impact of bedding volumes on laboratory mice. Appl Anim Behav Sci. (2017) 186:72–9. 10.1016/j.applanim.2016.11.004 29160176

[5] Latham N Mason G From house mouse to mouse house: the behavioural biology of free-living Mus musculus and its implications in the laboratory. Appl Anim Behav Sci. (2004) 86:261–89. 10.1016/j.applanim.2004.02.006

[6] Keenan KP Ballam CG Haught DG Laroque P Nutrition. In: Krinke GJ editors. The Laboratory Rat. Orlando, FL: Elsevier; Academic Press (2000). p. 57–76.

[7] Keenan KP Hoe C-M Mixson L Mccoy CL Coleman JB Mattson BA. Diabesity: a polygenic model of dietary-induced obesity from *ad libitum* overfeeding of Sprague–Dawley rats and its modulation by moderate and marked dietary restriction. Toxicol Pathol. (2005) 33:650–74. 10.1080/01926230500311222 16207639

[8] Keenan K Laroque P Soper K Morrissey R Dixit R. The effects of overfeeding and moderate dietary restriction on Sprague-Dawley rat survival, pathology, carcinogenicity, and the toxicity of pharmaceutical agents. Exp Toxicol Pathol. (1996) 48:139–44. 10.1016/S0940-2993(96)80034-0 8672867

[9] Roe F 1200-rat biosure study: desing and overview of results. In: Fishbein L editors. Biological Effects of Dietary Restriction. Berlin; Heidelberg: ILSI Monographs; Springer (1991) 287–304. 10.1007/978-3-642-58181-6_26

[10] Roe F Lee P Conybeare G Kelly D Matter B Prentice D. The biosure study: influence of composition of diet and food consumption on longevity, degenerative diseases and neoplasia in Wistar rats studied for up to 30 months post weaning. Food Chem Toxicol. (1995) 33:S1–100. 10.1016/0278-6915(95)80200-2 7713457

[11] Rowland NE Food or fluid restriction in common laboratory animals: balancing welfare considerations with scientific inquiry. Comp Med. (2007) 57:149–60. 17536615

[12] Toth LA Gardiner TW. Food and water restriction protocols: physiological and behavioral considerations. J Am Assoc Lab Anim Sci. (2000) 39:9–17. 11487246

[13] Young JW Sharkey J Finlayson K. Progressive impairment in olfactory working memory in a mouse model of mild cognitive impairment. Neurobiol Aging. (2009) 30:1430–43. 10.1016/j.neurobiolaging.2007.11.018 18242780

[14] Valentim AM Alves HCS Olsson IA Antunes LM. The effects of depth of isoflurane anesthesia on the performance of mice in a simple spatial learning task. J Am Assoc Lab Anim Sci. (2008) 47:16–9. 18459707 PMC2654004

[15] Krakenberg V Woigk I Rodriguez LG Kästner N Kaiser S Sachser N. Technology or ecology? New tools to assess cognitive judgement bias in mice. Behav Brain Res. (2019) 362:279–87. 10.1016/j.bbr.2019.01.021 30654122

[16] Magen I Fleming SM Zhu C Garcia EC Cardiff KM Dinh D. Cognitive deficits in a mouse model of pre-manifest Parkinson's disease. Eur J Neurosci. (2012) 35:870–82. 10.1111/j.1460-9568.2012.08012.x 22356593 PMC3967873

[17] Mallien AS Palme R Richetto J Muzzillo C Richter SH Vogt MA. Daily exposure to a touchscreen-paradigm and associated food restriction evokes an increase in adrenocortical and neural activity in mice. Hormones Behav. (2016) 81:97–105. 10.1016/j.yhbeh.2016.03.009 27059527

[18] Richter SH Vogel AS Ueltzhöffer K Muzzillo C Vogt MA Lankisch K. Touchscreen-paradigm for mice reveals cross-species evidence for an antagonistic relationship of cognitive flexibility and stability. Front Behav Neurosci. (2014) 8:154. 10.3389/fnbeh.2014.00154 24834036 PMC4017158

[19] Mattson MP. Energy intake, meal frequency, and health: a neurobiological perspective. Annu Rev Nutr. (2005) 25:237–60. 10.1146/annurev.nutr.25.050304.092526 16011467

[20] Duffy P Seng J Lewis S Mayhugh M Aidoo A Hattan D. The effects of different levels of dietary restriction on aging and survival in the Sprague-Dawley rat: implications for chronic studies. Aging Clin Exp Res. (2001) 13:263–72. 10.1007/BF03353422 11695495

[21] Blackwell B-N Bucci TJ Hart RW Turturro A. Longevity, body weight, and neoplasia in *ad libitum*-fed and diet-restricted C57BL6 mice fed NIH-31 open formula diet. Toxicol Pathol. (1995) 23:570–82. 10.1177/019262339502300503 8578100

[22] Sarkar NH Fernandes G Telang NT Kourides IA Good RA. Low-calorie diet prevents the development of mammary tumors in C3H mice and reduces circulating prolactin level, murine mammary tumor virus expression, and proliferation of mammary alveolar cells. Proc Natl Acad Sci USA. (1982) 79:7758–62. 10.1073/pnas.79.24.7758 6296850 PMC347427

[23] Weindruch R Walford RL Fligiel S Guthrie D. The retardation of aging in mice by dietary restriction: longevity, cancer, immunity and lifetime energy intake. J Nutr. (1986) 116:641–54. 10.1093/jn/116.4.641 3958810

[24] Keenan KP Smith PF Hertzog P Soper K Ballam GC Clark RL. The effects of overfeeding and dietary restriction on Sprague-Dawley rat survival and early pathology biomarkers of aging. Toxicol Pathol. (1994) 22:300–15. 10.1177/019262339402200308 7817120

[25] Hashimoto T Watanabe S. Chronic food restriction enhances memory in mice–analysis with matched drive levels. Neuroreport. (2005) 16:1129–33. 10.1097/00001756-200507130-00019 15973161

[26] Ingram DK Weindruch R Spangler EL Freeman JR Walford RL. Dietary restriction benefits learning and motor performance of aged mice. J Gerontol. (1987) 42:78–81. 10.1093/geronj/42.1.78 3794202

[27] Hohlbaum K Bert B Dietze S Palme R Fink H Thöne-Reineke C. Severity classification of repeated isoflurane anesthesia in C57BL/6JRj mice—Assessing the degree of distress. PLoS ONE. (2017) 12:e0179588. 10.1371/journal.pone.0179588 28617851 PMC5472303

[28] Bodden C Siestrup S Palme R Kaiser S Sachser N Richter SH. Evidence-based severity assessment: impact of repeated versus single open-field testing on welfare in C57BL/6J mice. Behav Brain Res. (2018) 336:261–8. 10.1016/j.bbr.2017.08.029 28842269

[29] Lewejohann L Kloke V Heiming RS Jansen F Kaiser S Schmitt A. Social status and day-to-day behaviour of male serotonin transporter knockout mice. Behav Brain Res. (2010) 211:220–8. 10.1016/j.bbr.2010.03.035 20347882

[30] Lepschy M Touma C Palme R. Faecal glucocorticoid metabolites: How to express yourself – comparison of absolute amounts versus concentrations in samples from a study in laboratory rats. Lab Anim. (2010) 44:192–8. 10.1258/la.2009.009082 20071410

[31] Touma C Palme R Sachser N. Analyzing corticosterone metabolites in fecal samples of mice: a noninvasive technique to monitor stress hormones. Hormones Behav. (2004) 45:10–22. 10.1016/j.yhbeh.2003.07.002 14733887

[32] Touma C Sachser N Möstl E Palme R. Effects of sex and time of day on metabolism and excretion of corticosterone in urine and feces of mice. General Comp Endocrinol. (2003) 130:267–78. 10.1016/S0016-6480(02)00620-2 12606269

[33] Palme R. Non-invasive measurement of glucocorticoids: Advances and problems. Physiol Behav. (2019) 199:229–43. 10.1016/j.physbeh.2018.11.021 30468744

[34] Brown LA Hasan S Foster RG Peirson SN. COMPASS: continuous open mouse phenotyping of activity and sleep status. Wellcome Open Res. (2017) 1:2–2. 10.12688/wellcomeopenres.9892.2 27976750 PMC5140024

[35] Foster RG Wulff K. The rhythm of rest and excess. Nat Rev Neurosci. (2005) 6:407. 10.1038/nrn1670 15861183

[36] Hastings MH Reddy AB Maywood ES. A clockwork web: circadian timing in brain and periphery, in health and disease. Nat Rev Neurosci. (2003) 4:649. 10.1038/nrn1177 12894240

[37] Mason GJ Stereotypies: a critical review. Anim Behav. (1991) 41:1015–37. 10.1016/S0003-3472(05)80640-2

[38] Mason GJ Latham NR Can't stop, won't stop: is stereotypy a reliable animal welfare indicator? Animal Welfare. (2004) 13(SUPPL.):S57–69.

[39] Martin P Bateson P Measuring Behaviour: An Introductory Guide. (2007) Cambridge: Cambridge University Press.

[40] Gouveia K Hurst JL. Reducing mouse anxiety during handling: effect of experience with handling tunnels. PLoS ONE. (2013) 8:e66401. 10.1371/journal.pone.0066401 23840458 PMC3688777

[41] Pellow S Chopin P File SE Briley M. Validation of open: closed arm entries in an elevated plus-maze as a measure of anxiety in the rat. J Neurosci Methods. (1985) 14:149–67. 10.1016/0165-0270(85)90031-7 2864480

[42] Lister RG. The use of a plus-maze to measure anxiety in the mouse. Psychopharmacology. (1987) 92:180–5. 10.1007/bf00177912 3110839

[43] Lister RG. Ethologically-based animal models of anxiety disorders. Pharmacol Ther. (1990) 46:321–40. 10.1016/0163-7258(90)90021-s 2188266

[44] Fuss J Richter SH Steinle J Deubert G Hellweg R Gass P. Are you real? Visual simulation of social housing by mirror image stimulation in single housed mice. Behav Brain Res. (2013) 243:191–8. 10.1016/j.bbr.2013.01.015 23333841

[45] Hurst JL West RS. Taming anxiety in laboratory mice. Nature Methods. (2010) 7:825. 10.1038/nmeth.1500 20835246

[46] Prior H Sachser N Effects of enriched housing environment on the behaviour of young male and female mice in four exploratory tasks. J Exp Anim Sci. (1995) 37:57–68.

[47] Chourbaji S Brandwein C Vogt MA Dormann C Hellweg R Gass P. Nature vs. nurture: Can enrichment rescue the behavioural phenotype of BDNF heterozygous mice? Behav Brain Res. (2008) 192:254–8. 10.1016/j.bbr.2008.04.015 18538870

[48] Ridder S Chourbaji S Hellweg R Urani A Zacher C Schmid W. Mice with genetically altered glucocorticoid receptor expression show altered sensitivity for stress-induced depressive reactions. J Neurosci. (2005) 25:6243–50. 10.1523/JNEUROSCI.0736-05.2005 15987954 PMC6725059

[49] Crawley J Goodwin FK. Preliminary report of a simple animal behavior model for the anxiolytic effects of benzodiazepines. Pharmacol Biochem Behav. (1980) 13:167–70. 10.1016/0091-3057(80)90067-2 6106204

[50] Archer J. Tests for emotionality in rats and mice: a review. Anim Behav. (1973) 21:205–35. 10.1016/S0003-3472(73)80065-X 4578750

[51] Treit D Fundytus M. Thigmotaxis as a test for anxiolytic activity in rats. Pharmacol Biochem Behav. (1988) 31:959–62. 10.1016/0091-3057(88)90413-3 3252289

[52] Hoyer C Vogt MA Richter SH Zaun G Zahedi Y Maderwald S Repetitive exposure to a 7 Tesla static magnetic field of mice in utero does not cause alterations in basal emotional and cognitive behavior in adulthood. Reprod Toxicol. (2012) 34:86–92. 10.1016/j.reprotox.2012.03.006 22484359

[53] Gaskill BN Karas AZ Garner JP Pritchett-Corning KR Nest building as an indicator of health and welfare in laboratory mice. J Vis Exp. (2013) 82:51012 10.3791/51012 PMC4108067 24429701

[54] Deacon RMJ. Assessing nest building in mice. Nat Protoc. (2006) 1:1117. 10.1038/nprot.2006.170 17406392

[55] R Core Team. R: A Language and Environment for Statistical Computing. Vienna: R Foundation for Statistical Computing. (2015). Available online at: https://www.R-project.org/ (accessed August 20, 2019).

[56] RStudio Team RStudio: Integrated Development for R. Boston, MA: RStudio. Inc (2016). Available online at: http://www.rstudio.com/ (accessed August 20, 2019).

[57] Faul F Erdfelder E Lang A-G Buchner A. G^*^ Power 3: a flexible statistical power analysis program for the social, behavioral, and biomedical sciences. Behav Res Methods. (2007) 39:175–91. 10.3758/BF03193146 17695343

[58] Lakens D. Calculating and reporting effect sizes to facilitate cumulative science: a practical primer for t-tests and ANOVAs. Front Psychol. (2013) 4:863. 10.3389/fpsyg.2013.00863 24324449 PMC3840331

[59] Cohen J Statistical Power Analysis for the Behavioral Sciences. 2nd Edn. New York, NY: Routledge (1988).

[60] Keenan KP Laroque P Ballam GC Soper KA Dixit R Mattson BA. The effects of diet, *ad libitum* overfeeding, and moderate dietary restriction on the rodent bioassay: the uncontrolled variable in safety assessment. Toxicol Pathol. (1996) 24:757–68. 10.1177/019262339602400620 8994307

[61] Paul ES Harding EJ Mendl M. Measuring emotional processes in animals: the utility of a cognitive approach. Neurosci Biobehav Rev. (2005) 29:469–91. 10.1016/j.neubiorev.2005.01.002 15820551

[62] Riley V. Psychoneuroendocrine influences on immunocompetence and neoplasia. Science. (1981) 212:1100–9. 10.1126/science.7233204 7233204

[63] Pfeiffenberger U Yau T Fink D Tichy A Palme R Egerbacher M. Assessment and refinement of intra-bone marrow transplantation in mice. Lab Anim. (2015) 49:121–31. 10.1177/0023677214559627 25416608

[64] Jansen F Heiming RS Lewejohann L Touma C Palme R Schmitt A. Modulation of behavioural profile and stress response by 5-HTT genotype and social experience in adulthood. Behav Brain Res. (2010) 207:21–9. 10.1016/j.bbr.2009.09.033 19782704

[65] Nicholson A Malcolm RD Russ PL Cough K Touma C Palme R. The response of C57BL/6J and BALB/cJ mice to increased housing density. J Am Assoc Lab Anim Sci. (2009) 48:740–53. 19930822 PMC2786928

[66] Mason G Stereotypic behaviour in captive animals: fundamentals and implications for welfare and beyond. Stereo Anim Behav. (2006) 2:325–56. 10.1079/9780851990040.0325

[67] Hadley C Hadley B Ephraim S Yang M Lewis MH Spontaneous stereotypy and environmental enrichment in deer mice (*Peromyscus maniculatus*): reversibility of experience. Appl Anim Behav Sci. (2006) 97:312–22. 10.1016/j.applanim.2005.08.006

[68] Powell SB Newman HA McDonald TA Bugenhagen P Lewis MH. Development of spontaneous stereotyped behavior in deer mice: effects of early and late exposure to a more complex environment. Dev Psychobiol. 37:100–8. 10.1002/1098-2302(200009)37:2<100::AID-DEV5>3.0.CO;2-6 10954835

[69] Ödberg F. The influence of cage size and environmental enrichment on the development of stereotypies in bank voles (*Clethrionomys glareolus*). Behav Proc. (1987) 14:155–73. 10.1016/0376-6357(87)90042-8 24897258

[70] Abou-Ismail U Burman O Nicol C Mendl M Can sleep behaviour be used as an indicator of stress in group-housed rats (*Rattus norvegicus*)? Anim Welfare. (2007) 16:185–8.

[71] Krieger DT. Food and water restriction shifts corticosterone, temperature, activity and brain amine periodicity. Endocrinology. (1974) 95:1195–201. 10.1210/endo-95-5-1195 4426285

[72] Gooley JJ Schomer A Saper CB. The dorsomedial hypothalamic nucleus is critical for the expression of food-entrainable circadian rhythms. Nat Neurosci. (2006) 9:398–407. 10.1038/nn1651 16491082

[73] Stephan FK. The “other” circadian system: food as a Zeitgeber. J Biol Rhythms. (2002) 17:284–92. 10.1177/074873040201700402 12164245

[74] Genn RF Tucci SA Thomas A Edwards JE File SE. Age-associated sex differences in response to food deprivation in two animal tests of anxiety. Neurosci Biobehav Rev. (2003) 27:155–61. 10.1016/S0149-7634(03)00017-4 12732231

[75] Makowiecki K Hammond G Rodger J. Different levels of food restriction reveal genotype-specific differences in learning a visual discrimination task. PLoS ONE. (2012) 7:e48703. 10.1371/journal.pone.0048703 23144936 PMC3492417

